# Hospital admissions linked to SARS-CoV-2 infection in children and adolescents: cohort study of 3.2 million first ascertained infections in England

**DOI:** 10.1136/bmj-2022-073639

**Published:** 2023-07-05

**Authors:** Harrison Wilde, Christopher Tomlinson, Bilal A Mateen, David Selby, Hari Krishnan Kanthimathinathan, Padmanabhan Ramnarayan, Pascale Du Pre, Mae Johnson, Nazima Pathan, Arturo Gonzalez-Izquierdo, Alvina G Lai, Deepti Gurdasani, Christina Pagel, Spiros Denaxas, Sebastian Vollmer, Katherine Brown

**Affiliations:** 1 Department of Statistics, University of Warwick, Warwick, UK; 2 University College London (UCL) Institute of Health Informatics, UCL, London, UK; 3 UCL UK Research and Innovation Centre for Doctoral Training in AI-enabled Healthcare Systems, UCL, London, UK; 4 University College London Hospitals Biomedical Research Centre, UCL, London, UK; 5 Wellcome Trust, London, UK; 6 Department for Data Science and its Applications, German Research Centre for Artificial Intelligence (DFKI), Kaiserslautern, Germany; 7 Department of Computer Science, TU Kaiserslautern, Kaiserslautern, Germany; 8 Paediatric Intensive Care Unit, Birmingham Women’s and Children’s NHS Foundation Trust, Birmingham, UK; 9 Department of Surgery and Cancer, Faculty of Medicine, Imperial College London, London UK Imperial College London, London, UK; 10 Biomedical Research Centre, Great Ormond Street Hospital for Children, London, UK; 11 University Department of Paediatrics, Cambridge University, Cambridge, UK; 12 William Harvey Institute, Queen Mary University of London, London, UK; 13 Kirby Institute, University of New South Wales, Sydney, NSW, Australia; 14 Clinical Operational Research Unit, UCL, London, UK; 15 Institute of Cardiovascular Science, UCL, London, UK

## Abstract

**Objective:**

To describe hospital admissions associated with SARS-CoV-2 infection in children and adolescents.

**Design:**

Cohort study of 3.2 million first ascertained SARS-CoV-2 infections using electronic health care record data.

**Setting:**

England, July 2020 to February 2022.

**Participants:**

About 12 million children and adolescents (age <18 years) who were resident in England.

**Main outcome measures:**

Ascertainment of a first SARS-CoV-2 associated hospital admissions: due to SARS-CoV-2, with SARS-CoV-2 as a contributory factor, incidental to SARS-CoV-2 infection, and hospital acquired SARS-CoV-2.

**Results:**

3 226 535 children and adolescents had a recorded first SARS-CoV-2 infection during the observation period, and 29 230 (0.9%) infections involved a SARS-CoV-2 associated hospital admission. The median length of stay was 2 (interquartile range 1-4) days) and 1710 of 29 230 (5.9%) SARS-CoV-2 associated admissions involved paediatric critical care. 70 deaths occurred in which covid-19 or paediatric inflammatory multisystem syndrome was listed as a cause, of which 55 (78.6%) were in participants with a SARS-CoV-2 associated hospital admission. SARS-CoV-2 was the cause or a contributory factor in 21 000 of 29 230 (71.8%) participants who were admitted to hospital and only 380 (1.3%) participants acquired infection as an inpatient and 7855 (26.9%) participants were admitted with incidental SARS-CoV-2 infection. Boys, younger children (<5 years), and those from ethnic minority groups or areas of high deprivation were more likely to be admitted to hospital (all P<0.001). The covid-19 vaccination programme in England has identified certain conditions as representing a higher risk of admission to hospital with SARS-CoV-2: 11 085 (37.9%) of participants admitted to hospital had evidence of such a condition, and a further 4765 (16.3%) of participants admitted to hospital had a medical or developmental health condition not included in the vaccination programme’s list.

**Conclusions:**

Most SARS-CoV-2 associated hospital admissions in children and adolescents in England were due to SARS-CoV-2 or SARS-CoV-2 was a contributory factor. These results should inform future public health initiatives and research.

## Introduction

Severe disease related to SARS-CoV-2 infection is rare in children and adolescents, with reported rates for hospital admissions of 300-450 per 100 000 ascertained SARS-CoV-2 infections (Germany^1^) and reported case fatality rates of 0.7-0.9 per 100 000 ascertained SARS-CoV-2 infections (UK, Italy^2^ ^3^). These lower rates of severe disease can be linked to reported lesser intrinsic severity of SARS-CoV-2 infection in younger age groups.^4^ Between the first reported SARS-CoV-2 infection in England at the end of January 2020, leading to the original variant wave, and the summer of 2022, there were seven further peaks in SARS-CoV-2 prevalence.^5^ ^6^ From September 2021 to April 2022, the Office for National Statistics survey reported that the highest rates of SARS-CoV-2 infections of all age groups was in children and adolescents.^5^ These high levels of infection led to an increase in paediatric hospital admissions in the second year of the pandemic compared with the first year,^7^ in contrast with adults where hospital admissions decreased, largely related to the high rates of vaccination, particularly for people classed as vulnerable.^8^ Nonetheless evidence is lacking among children and adolescents admitted to hospital about the severity of illness; the rates of SARS-CoV-2 related illness, rather than admissions where the infection either was incidental or was acquired in hospital; the evolving epidemiology of covid-19 across different variant epochs; and to what extent those without underlying health conditions experience severe disease with SARS-CoV-2 infection. The last was particularly pivotal to the delay between approval of vaccines by the medicines regulator (Medicines and Healthcare Products Regulatory Agency) and approval for use, because the risks of hospital admission were considered minimal in children and young people without underlying health conditions.^9^ ^10^ ^11^ We present a comprehensive analysis of all SARS-CoV-2 associated hospital admissions among the estimated 12 million children and adolescents younger than 18 years resident in England (ONS^12^) during the period from when the community testing programme had commenced^13^ (1 July 2020) until the most recent available data (31 March 2022). In this study we classify paediatric hospital admissions linked to SARS-CoV-2 infection as those primarily due to SARS-CoV-2 infection; those where SARS-CoV-2 was likely to be on the causal pathway; those incidental to infection; and those that were acquired in hospital (nosocomial). We also describe the personal characteristics and underlying health conditions among children and adolescents with a first SARS-CoV-2 infection by hospital admission type; and the trends in first ascertained SARS-CoV-2 infections, hospital admissions, intensive care unit (ICU) admissions, and SARS-CoV-2 infection associated hospital admission rates stratified by dominant variant eras (original, alpha, delta, and omicron).

## Methods

### Design and data sources

In this national, retrospective cohort study based on routinely collected, electronic health record data, we used NHS England’s trusted research environment for England accessed through the British Heart Foundation Data Science Centre’s CVD-COVID-UK/COVID-IMPACT consortium^14^ to create a linked cohort comprising the following datasets: National laboratory covid-19 testing data from the Public Health England (now the UK Health Security Agency) Second Generation Surveillance System; primary care data from the General Practice Extraction Service Data for Pandemic Planning and Research; and Hospital Episode Statistics, including admitted patient care, critical care, and outpatient data. HES critical care data identifies patients admitted to hospital who are coded as fulfilling the Paediatric Critical Care Minimum Data Set,^15^ which provides a record of what happens to patients when they receive paediatric critical care in an intensive care unit (ICU) or high dependency unit (HDU); deaths from the ONS Civil Registration of Death, including the causes of death listed in order^16^; and covid-19 vaccination status from NHS England.

Datasets were linked by NHS England using the NHS number, a unique persistent healthcare identifier assigned at first encounter with the healthcare system.^14^ We analysed the data as presented, leveraging this linkage to ensure the presence of important characteristics (eg, date of birth, admission date) if one was present across any of the relevant records, but otherwise carried out no imputation when data were missing. We addressed missing variables and conducted a complete case analysis.

### Inclusion criteria and study cohort

Children and adolescents were considered eligible for inclusion in the study if they were aged 0-17 years at the time of first ascertained SARS-CoV-2 infection, were resident in England, had a valid person pseudo-identifier enabling data linkage, were alive at study start or born during the study period, and their sex was known.

We created a cohort of children and adolescents who had a first ascertained SARS-CoV-2 infection during the study period of 1 July 2020 to 17 February 2022. First ascertained infections were identified based on either a first positive SARS-CoV-2 test result in the Second Generation Surveillance System, or a first SARS-CoV-2 associated hospital admission. When applicable, we linked participants’ first positive test result with their first SARS-CoV-2 associated hospital admission if the test occurred between six weeks before the date of admission (the maximum reported time between an infection and hospital admission for paediatric inflammatory multisystem syndrome^17^) and the date of hospital discharge (for identification of nosocomial infections). We allowed six weeks follow-up for infections identified by positive test results, to capture SARS-CoV-2 associated admissions occurring up to 31 March 2022, for the purposes of calculating the rate of SARS-CoV-2 related hospital admissions. We excluded reinfections and second admissions related to SARS-CoV-2—we considered the analysis of reinfections to be a separate, complex topic beyond the scope of this study.

### Outcome: SARS-CoV-2 associated hospital admission and admission type

Because SARS-CoV-2 is a relatively new virus with emerging clinical characteristics in children and adolescents, we used broad inclusion criteria to identify SARS-CoV-2 associated hospital admissions, before classifying the admissions by type (box 1; also see supplementary table A). Basing our approach on formative NHS based research in children and adolescents with SARS-CoV-2,^10^ ^11^ we included first SARS-CoV-2 associated hospital admissions where at least one of the following criteria was met: from HES Admitted Patient Care, a primary cause for hospital admission was one of ICD-10 (international classification of diseases, 10th revision) codes U07.1, U07.2, U07.3, or U07.4, or a non-primary cause for hospital admission was U07.1 or U07.2^11^; from HES Admitted Patient Care, a primary or non-primary cause for admission an ICD-10 code used to identify paediatric inflammatory multisystem syndrome (introduced from May 2020): R65, M30.3, or, from November 2020, U07.5,^11^ and no exclusion codes were present that indicated an alternative diagnosis; or there was a positive SARS-CoV-2 test result from up to 14 days before hospital admission until the date of hospital discharge.^18^

Box 1 **List of hospital admission types in order of assignment for categorising SARS-CoV-2 associated hospital admissions, using hierarchical criteria**

**Nosocomial SARS-CoV-2 infection**

Consistent with definitions used by NHS England,^18^ admissions were classified as nosocomial if the first associated positive SARS-CoV-2 test result occurred between day 8 of hospital admission and hospital discharge and there were no covid-19 codes (U07.1, U07.2) provided as a cause for hospital admission before day 8 of the admission.

**Type C: Incidental SARS-CoV-2 infection**

Admissions judged to be incidental to SARS-CoV-2 infection were identified before those due to SARS-CoV-2 or SARS-CoV-2 was a contributory factor, to avoid misclassification. These are admissions where SARS-CoV-2 is not the cause but is coincidental as a result of community transmission. Incidental admissions all had codes U07.1 or U07.2 as a non-primary reason for admission or a positive test result before day 8 of the admission, or both. Candidate reasons were identified in the ISARIC prospective study of covid-19 in children, such as trauma, poisoning, or elective surgery.^19^ A wider range of relevant primary reasons for admission than used by ISARIC was identified and included by iterative clinical review of codes present in the hospital admission dataset: mental health disorders, eye conditions, dental conditions, injuries, trauma, assault, self-harm, poisoning, surgical problems such as those affecting bowel or testis, and certain pregnancy related conditions.

**Paediatric inflammatory multi system syndrome (PIMS-TS)**

Admissions were identified as due to PIMS-TS if a reason for hospital admission was PIMS-TS code U07.5, or a paediatric inflammatory multisystem syndrome code R65 or M30.3^11^ AND no reason for admission was a prespecified exclusion code indicating an alternative diagnosis of sepsis, specified bacterial or viral infections, or known causes of systemic inflammatory response. Patients admitted to hospital with PIMS-TS could also have a covid-19 code as a reason for admission and may or may not have a positive SARS-CoV-2 test result, as it has previously been shown that most are polymerase chain reaction negative at onset of PIMS-TS.^20^ ^21^ ^22^ After clinical review of the ICD-10 codes listed as the reasons for admission among children who had a PIMS-TS code as a non-primary reason for admission, these were included because (after applying the listed exclusion codes) these codes were consistent with a diagnosis of PIMS-TS.

**Type A: Caused by or suspected caused by SARS-CoV-2 infection**

Admissions caused by or suspected caused by SARS-CoV-2 infection.

Type A1—Primary reason for hospital admission was identified by one of four covid-19 codes: U07.1, U07.2, U07.3, or U07.4.^11^

Type A2—Primary reason for hospital admission was a sign, symptom, or condition or presentation consistent with an acute illness with SARS-CoV-2 infection (and did not definitively indicate an alternative diagnosis); AND a non-primary reason for hospital admission was a covid-19 code (U07.1 or U07.2) AND there was no excluded code indicating a reason for hospital admission was an alternative or co-infection. The candidate list of signs, symptoms, and conditions of SARS-CoV-2 infection was identified from prospective studies, including ISARIC and international studies^19^ ^23^ ^24^ ^25^ ^26^ and includes: unspecified viral infections, viral conjunctivitis, volume depletion, shock, acidosis, otitis media, croup, non-specific bronchiolitis, cough, fever, vomiting, diarrhoea, myalgia, headache, certain types of arrhythmias, tonsilitis, pharyngitis, and laryngitis. The candidate code list was checked iteratively against primary reasons for hospital admission in the cohort admitted to hospital, to generate the final code list. These admissions may or may not have been linked to a positive SARS-CoV-2 test result.

**Type B: SARS-CoV-2 infection as likely contributory factor**

Admissions where SARS-CoV-2 infection was likely to be on the causal pathway, albeit not the primary cause of the admission. These hospital admissions may or may not have been linked to a positive SARS-CoV-2 test result, and all these patients had a covid-19 code (U07.1 or U07.2) as a non-primary reason for hospital admission, combined with one of the following primary reasons for admission that were all deemed relevant based on published reports^19^ ^23^ ^27^ ^28^ ^29^ or, given the emerging nature of the topic, based on expert clinical experience:

Type B1—A condition known to co-occur with SARS-CoV-2 infection (co-infections or secondary infections due to, for example, respiratory syncytial virus, parainfluenza, adenovirus, staphylococcal pneumonia, streptococcal pneumonia)^27^ ^28^ ^29^; a condition that has been clinically linked to SARS-CoV-2 infection in children and adolescents^28^ ^29^ (type 1 diabetes mellitus, status epilepticus, or febrile seizures); or a small number of treatments that could be linked to SARS-CoV-2 infection (isolation in cubicle for droplet precautions)

Type B2—A pre-existing or newly diagnosed condition associated with higher risk of severe illness with SARS-CoV-2 infection^19^ ^23^ (conditions treated with immunosuppressants, any cancer, neurodevelopmental conditions that may affect breathing, neonatal conditions such as poor feeding, respiratory diseases such as asthma)

ISARIC=International Severe Acute Respiratory and emerging Infection Consortium; PIMS-TS=paediatric inflammatory multisystem syndrome with a temporal association with SARS-CoV-2.For each patient in whom at least one Hospital Episode Statistics Admitted Patient Care consultant episode met one of the current study’s cohort inclusion criteria, all the episodes were grouped within the relevant hospital admission and all ICD-10 codes recorded as a reason for admission were collated, whether primary or non-primary. The ICD-10 codes as reason for admission were then used to identify hospital admission types.Definition of codes: U07.1=covid-19 with virus identified; U07.2=covid-19 with virus not identified; U07.3=personal history of covid-19; U07.4=post-covid-19 condition.Definition of paediatric inflammatory multisystem syndrome codes: R65=systemic inflammatory response; M30.3=Kawasaki disease; U07.5=paediatric inflammatory multisystem syndrome.Supplementary table A provides detailed code descriptions and lists for each hospital admission type.

From among first SARS-CoV-2 associated hospital admissions, we used a hierarchical approach to identify mutually exclusive admission types, using a combination of the ICD-10 codes listed as a reason for admission in HES Admitted Patient Care records, and positive SARS-CoV-2 test results in the Second Generation Surveillance System testing data. For clinical reasons developed by consensus, SARS-CoV-2 associated hospital admission types were identified in the order: admissions with nosocomial infection; admissions with incidental infection; admissions with paediatric inflammatory multisystem syndrome hospital; admissions due to or suspected to be due to SARS-CoV-2 infection (not paediatric inflammatory multisystem syndrome); and admissions where SARS-CoV-2 infection was a contributory factor (not paediatric inflammatory multisystem syndrome). The consultant paediatrician panel, all of whom have direct experience of caring for children and adolescents admitted to hospital with SARS-CoV-2 (KB, HKK, NP, MJ, PDP, PR) contributed to the methods, and at least two members had to agree on each clinical code.

### Personal characteristics

We extracted date of birth (this was used to calculate age at date of infection; grouped as <1, 1-4, 5-11, 12-15, and 16-17 years), sex, ethnicity (coded through adapted ONS census categories and the HES Admitted Patient Care data dictionary: Asian or Asian British, black ethnicity or black British, Chinese, mixed, other, unknown, white ethnicity), and information on lower layer super output areas from primary and secondary care records. Information on socioeconomic deprivation was derived by mapping patients’ lower layer super output areas to the English index of multiple deprivation; these were then reported as fifths.

### Underlying health conditions

We identified medical and developmental underlying health conditions given their importance as risk factors for severe disease with SARS-CoV-2.^23^ ^30^ ^31^ ^32^ Supplementary table B provides details of pre-existing health conditions. Firstly, clinicians cross referenced all conditions listed in Chapter 14a of the Green Book^33^ against ICD-10^34^ to identify codes representing the conditions that were flagged as leading to clinical vulnerability with SARS-CoV-2 infection by the Joint Committee on Vaccination and Immunisation. Secondly, clinicians considered reference sources of ICD-10^34^ and a scheme for identifying comorbidities in HES by Hardelid and colleagues^35^ specific to children and adolescents, to create a code list of additional candidate underlying health conditions. The HES Admitted Patient Care ICD-10 codes present within the SARS-CoV-2 associated hospital admissions were reviewed by clinicians, underlying health conditions were identified, and acute conditions due to SARS-CoV-2 were removed from the list of underlying health conditions. Hence, iteratively, informed by clinician review and exploratory data analysis, a more inclusive list of medical and developmental underlying health conditions was created. For children, given their young ages a first hospital admission might be the only source of information on underlying health conditions, hence we considered any mention of congenital or chronic underlying health conditions in the electronic health record from birth to first ascertained infection, inclusive (this differs from the method used in adults, where only the history is considered^36^ ^37^). In acquired or reversible underlying health conditions (eg, cancer, conditions of prematurity), as undertaken in previous studies of underlying health conditions in children by Hardelid and colleagues,^35^ ^38^ we limited these to five years up to and including the first ascertained infection. We then sought the selected ICD-10 codes in General Practice Extraction Service Data for Pandemic Planning and Research (primary care), and additional HES Admitted Patient Care and HES Outpatient (secondary care) records, dated up to and including the admission of interest. In the case of General Practice Extraction Service Data for Pandemic Planning and Research, we transformed SNOMED-CT codes into ICD-10 codes using NHS England’s Technology Reference Update Distribution cross maps.^39^

Given previous evidence that obesity is linked to severe covid-19,^23^ ^30^ we extracted the weights and heights of participants from the General Practice Extraction Service Data for Pandemic Planning and Research in the two years before the first ascertained infection, and calculated body mass index (BMI) when both values were present. Reference data were used to calculate a BMI, or where there was no height, weight-for-age and sex standard deviation score^40^; we then defined a binary indicator for obesity based on World Health Organization criteria (younger than 5 years: BMI or weight-for-age z scores >3, older than 5 years: BMI or weight-for-age z scores >2).^41^ Finally, based on electronic health record codes from the nine months before the first ascertained infection—or codes from the current admission when applicable, we identified those in the cohort who were pregnant.

### Time eras for SARS-CoV-2 variants

We assigned a dominant SARS-CoV-2 variant to each infection in the cohort using the following time eras: original variant—1 July 2020 to 5 December 2020; alpha variant—3 January 2021 to 1 May 2021; delta variant—30 May 2021 to 11 December 2021; omicron variant—26 December to study end (17 February 2022 for first ascertained infections and 31 March for admissions meeting our criteria where an infection occurred before 17 February 2022).^42^ To avoid periods where two variants crossed over, we defined inter-variant periods between each of the time era windows.^42^

### Vaccination status

Vaccination status was coded as one of unvaccinated or first, second, or third (booster) dose (vaccinated) and was determined by the number of vaccinations received 14 days before infection. Vaccine type was not reported, as only the Pfizer-BioNTech covid-19 vaccine (Comirnaty) is approved for use in children and adolescents in England.

### Statistical analysis

#### Descriptive statistics

Among the cohort of children and adolescents with a first ascertained SARS-CoV-2 infection, we compared prespecified variables among those infections that did or did not involve a SARS-CoV-2 associated hospital admission using χ^2^ tests. We describe the severity of the SARS-CoV-2 infection associated hospital admission by length of stay and the proportion of participants admitted to an ICU or HDU based on the Paediatric Critical Care Minimum Data Set.^15^ As specified by the Joint Committee on Vaccination and Immunisation,^43^ ^44^ we defined a severe SARS-CoV-2 related hospital admission as either an ICU admission or admission due to paediatric inflammatory multisystem syndrome (whether or not it involved ICU). We ascertained deaths among children and adolescents with a first ascertained SARS-CoV-2 infection at any time during the study period, categorised by whether the death occurred during the SARS-CoV-2 associated hospital admission and whether covid-19 or paediatric inflammatory multisystem syndrome was listed as a cause.

#### SARS-CoV-2 associated hospital admission rates

To calculate the rate of hospital admissions in participants with SARS-CoV-2 infection (the total and different types of hospital admission), we defined the denominator and numerator as all first ascertained infection episodes (one for each child) and the number of first infection episodes that contained a SARS-CoV-2 related first hospital admission, respectively.

### Patient and public involvement

The Patient and Public Oversight Panel of the CVD-COVID-UK/COVID-IMPACT consortium reviewed the protocol on 15 September 2021 and expressed strong support for research into direct impacts of SARS-CoV-2 infection on children’s and adolescent’s health. Two families of children admitted to hospital with SARS-CoV-2 kindly agreed to undertake a semi-structured interview about their experiences. We have liaised with representatives of the parent organisations Parents Utd, SafeEdForAll, LongCovidKids, OneVoice, ShieldUs, Clinically Vulnerable Families UK, and the Hazards Campaign. Comments on patient experiences from all these sources informed our selection of admission types (see box 1).

## Results

### Cohort and hospital admission types

**Fig 1:**
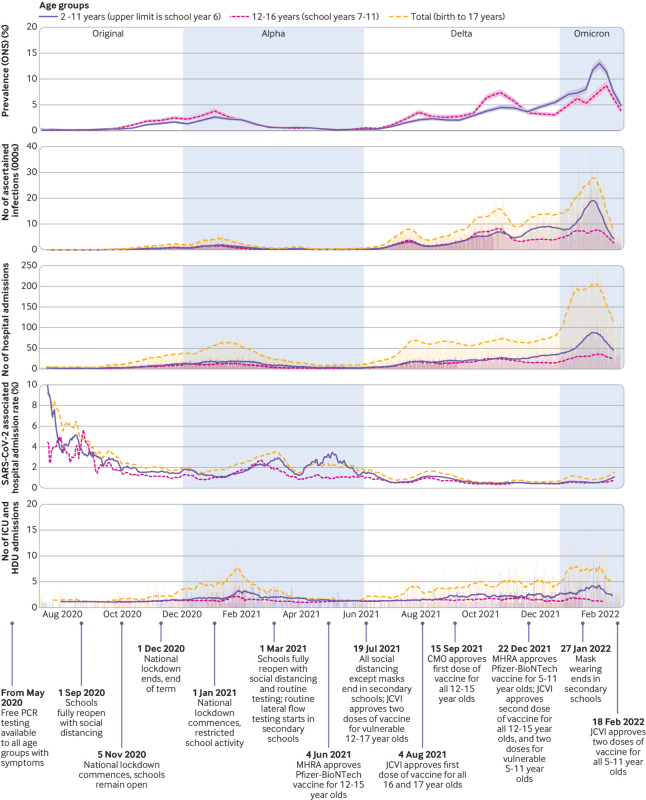
Key events linked to SARS-CoV-2 infections in children and adolescents during study period July 2020 to February 2022. From top panel to bottom panel: SARS-CoV-2 prevalence in children and adolescents reported by ONS survey; number of first ascertained SARS-CoV-2 infections derived from the current study data; number of first ascertained SARS-CoV-2 associated hospital admissions derived from current study data; hospital admission rate of participants with SARS-CoV-2 infection calculated using prevalence of ascertained first infections derived from current study data; number of first ascertained SARS-CoV-2 associated hospital admissions requiring ICU or HDU care. Top panel presents according to ONS data availability and reported confidence intervals (shaded areas); the other panels show 14 day rolling averages of daily counts. CMO=Chief Medical Officer; HDU=high dependency unit; ICU=intensive care unit; JCVI=Joint Committee on Vaccination and Immunisation; MHRA=Medicines and Healthcare Products Regulatory Agency; ONS=Office for National Statistics; PCR=polymerase chain reaction

During the study period, 3 226 535 first SARS-CoV-2 infections were ascertained in children and adolescents, of which 29 230 (0.9%) involved a first SARS-CoV-2 associated hospital admission, leaving 3 197 305 (99.1%) who were not admitted to hospital (fig 1). Table 1 provides detailed descriptive characteristics of the cohort. Among participants admitted to hospital, 1710 (5.9%) required ICU or HDU care (see supplementary table C and figure A). Among the full cohort of 3 226 535 participants with ascertained first infections, 70 deaths occurred in which either covid-19 or paediatric inflammatory multisystem syndrome was listed as a cause. The case fatality rate, inclusive of deaths where SARS-CoV-2 was one of the causes (ie, either causal or contributory) among first ascertained infections, was 2.2 per 100 000 ascertained infections. Of the 70 deaths, 55 occurred in participants with a SARS-CoV-2 associated hospital admission, representing 0.2% (55/29 230) of those with a first SARS-CoV-2 related hospital admission.

**Table 1:** Characteristics of cohort for all first ascertained SARS-CoV-2 infections in children and adolescents and by hospital admission type. Values are number (percentage) unless stated otherwise

Characteristics	First ascertained SARS-CoV-2 infections				Admission type						
	Total	Positive SARS-CoV-2 test result only	SARS-CoV-2 associated hospital admission		Nosocomial	Type C (incidental)	Paediatric inflammatory multisystem syndrome	Type A1 (caused by SARS-CoV-2 )	Type A2 (suspected caused by SARS-CoV-2)	Type B1 (causal pathway)	Type B2 (causal pathway)
Total No	3 226 535	3 197 305	29 230		380	7855	1790	9875	5330	2005	2000
**Sex**											
Female	1 618 030 (50.1)	1 604 175 (50.2)	13 855 (47.4)		245 (64.5)	4000 (50.9)	715 (39.9)	4640 (47.0)	2465 (46.2)	865 (43.1)	920 (46.0)
Male	1 608 505 (49.9)	1 593 130 (49.8)	15 375 (52.6)		135 (35.5)	3855 (49.1)	1075 (60.1)	5235 (53.0)	2860 (53.7)	1135 (56.6)	1080 (54.0)
**Age**											
Median (interquartile range) age (years)	11.4 (8.1-14.4)	11.5 (8.1-14.4)	5.5 (0.6-12.9)		7.4 (0.7-16.0)	10.6 (3.5-15.0)	7.6 (3.8-11.3)	1.3 (0.2-10.1)	4.1 (1.1-11.3)	1.2 (0.2-10.4)	9.5 (4.6-14.1)
Age group (years):											
<1	41 360 (1.2)	32 940 (1.0)	8415 (28.7)		120 (31.5)	1190 (15.2)	90 (5.1)	4590 (46.6)	1265 (23.7)	960 (47.8)	210 (10.4)
1-4	273 785 (8.5)	268 070 (8.4)	5720 (19.6)		65 (17.1)	1150 (14.6)	505 (28.2)	1770 (17.9)	1610 (30.2)	290 (14.5)	330 (16.5)
5-11	1 461 130 (45.3)	1 454 290 (45.5)	6840 (23.4)		20 (5.3)	2100 (26.7)	840 (46.9)	1545 (15.6)	1265 (23.7)	350 (17.5)	715 (35.8)
12-15	1 044 335 (32.4)	1 039 080 (32.5)	5255 (18.0)		85 (22.4)	2125 (27.1)	305 (17.0)	1225 (12.4)	765 (14.4)	255 (12.7)	495 (24.8)
16-17	405 925 (12.6)	402 925 (12.6)	3000 (10.3)		90 (23.7)	1290 (16.4)	50 (2.8)	745 (7.5)	425 (8.0)	150 (7.5)	250 (12.5)
**Ethnic group**											
Asian or Asian British	274 870 (8.5)	270 510 (8.5)	4360 (15.0)		40 (10.5)	1000 (12.8)	355 (19.9)	1570 (16.0)	750 (14.1)	290 (14.5)	345 (17.2)
Black or black British	87 725 (2.7)	85 865 (2.7)	1860 (6.4)		25 (6.6)	435 (5.5)	235 (13.1)	565 (5.7)	245 (4.6)	135 (6.7)	220 (11.0)
Chinese	16 350 (0.5)	16 250 (0.5)	100 (0.3)		0 (0.0)	25 (0.3)	15 (0.8)	40 (0.4)	15 (0.3)	<10 (<0.5)	<10 (<0.5)
Mixed	127 880 (4.0)	126 325 (4.0)	1555 (5.3)		25 (6.6)	380 (4.8)	130 (7.3)	555 (5.6)	260 (4.9)	110 (5.5)	90 (4.5)
Other	59 990 (1.9)	59 050 (1.8)	940 (3.2)		<10 (<2.6)	225 (2.9)	65 (3.6)	360 (3.6)	160 (3.0)	70 (3.5)	55 (2.8)
Unknown	36 915 (1.1)	36 685 (1.1)	235 (0.8)		<10 (<2.6)	55 (0.7)	15 (0.8)	85 (0.9)	50 (0.9)	15 (0.7)	15 (0.8)
White	2 622 805 (81.3)	2 602 620 (81.4)	20 180 (69.0)		275 (72.4)	5735 (73.0)	975 (54.5)	6700 (67.8)	3850 (72.2)	1380 (68.8)	1265 (63.2)
**Index of multiple deprivation fifth**											
1st (most deprived)	625 285 (19.3)	616 440 (19.3)	8845 (30.3)		95 (25.0)	2515 (32.0)	480 (26.8)	2945 (29.8)	1525 (28.7)	635 (31.6)	645 (32.1)
2nd	592 080 (18.4)	585 575 (18.3)	6505 (22.3)		100 (26.3)	1685 (21.5)	395 (22.1)	2195 (22.2)	1205 (22.6)	475 (23.7)	455 (22.8)
3rd	622 550 (19.3)	617 370 (19.3)	5180 (17.7)		75 (19.7)	1315 (16.7)	335 (18.7)	1820 (18.4)	940 (17.6)	365 (18.2)	335 (16.8)
4th	654 815 (20.3)	650 160 (20.3)	4655 (15.9)		60 (15.8)	1230 (15.7)	305 (17.0)	1595 (16.2)	880 (16.5)	280 (14.0)	310 (15.5)
5th (least deprived)	731 805 (22.7)	727 760 (22.8)	4045 (13.8)		50 (13.2)	1110 (14.1)	275 (15.4)	1320 (13.4)	780 (14.6)	250 (12.5)	255 (12.8)
**Underlying health conditions**											
Health condition linked to clinical vulnerability	580 195 (18.0)	569 110 (17.8)	11 085 (37.9)		210 (55.3)	2550 (32.5)	515 (28.8)	3190 (32.3)	1730 (32.5)	1140 (56.9)	1745 (87.2)
Medical and developmental condition	939 965 (29.1)	923 690 (28.9)	16 275 (55.7)		280 (73.7)	3880 (49.4)	1020 (57.0)	4815 (48.8)	2795 (52.4)	1500 (74.8)	1990 (99.5)
**Hospital admission features **											
Covid-19 positivity in window*	-	-	23 750 (81.3)		380 (100.0)	7005 (89.2)	130 (7.3)	8135 (82.4)	4615 (86.6)	1695 (84.5)	1795 (89.8)
Length of stay	-	-	2 (1 4)		93 (42 162)	2 (1 4)	6 (4 8)	2 (1 3)	2 (1 2)	3 (2 6)	2 (1 3)
Still in hospital	-	-	160 (0.5)		95 (25.0)	40 (0.5)	0 (0.0)	<10 (<0.1)	<10 (<0.2)	15 (0.7)	<10 (<0.5)
ICU or HDU care	-	-	1710 (5.9)		105 (27.6)	255 (3.2)	535 (29.9)	315 (3.2)	95 (1.8)	320 (16.0)	85 (4.2)
Day patient	-	-	1805 (6.2)		0 (0.0)	815 (10.4)	85 (4.7)	70 (0.7)	95 (1.8)	75 (3.7)	665 (33.2)
**Vaccination status 14 days before infection**											
Unvaccinated	2 878 355 (89.2)	2 850 515 (89.1)	27 840 (95.2)		340 (89.5)	7290 (92.8)	1760 (98.3)	9605 (97.2)	5115 (96.0)	1925 (96.0)	1805 (90.2)
First dose	297 030 (9.2)	296 010 (9.3)	1020 (3.5)		30 (7.9)	445 (5.7)	25 (1.4)	180 (1.8)	160 (3.0)	55 (2.7)	125 (6.2)
Second dose	48 875 (1.5)	48 560 (1.5)	315 (1.1)		<10 (<2.6)	110 (1.4)	<10 (<0.6)	65 (0.7)	45 (0.8)	25 (1.2)	60 (3.0)
Booster dose	2275 (0.1)	2220 (0.1)	55 (0.2)		<10 (<2.6)	10 (0.1)	0 (0.0)	25 (0.3)	<10 (<0.2)	<10 (<0.5)	<10 (<0.5)
**Variant period**											
Original	127 205 (3.8)	124 770 (3.8)	2450 (8.3)		35 (9.2)	730 (9.4)	230 (12.8)	690 (6.9)	430 (8.1)	155 (7.8)	175 (8.7)
Original-alpha	101 885 (3.2)	100 435 (3.1)	1450 (5.0)		35 (9.2)	410 (5.2)	105 (5.9)	445 (4.5)	240 (4.5)	120 (6.0)	95 (4.7)
Alpha	158 960 (4.9)	155 660 (4.9)	3300 (11.3)		60 (15.8)	895 (11.4)	425 (23.7)	945 (9.6)	575 (10.8)	185 (9.2)	210 (10.4)
Alpha-delta	15 525 (0.5)	15 260 (0.5)	260 (0.9)		<10 (<2.6)	65 (0.8)	35 (2.0)	75 (0.8)	50 (0.9)	25 (1.2)	15 (0.8)
Delta	1 612 630 (50.0)	1 601 445 (50.1)	11 185 (38.3)		95 (25.0)	2945 (37.5)	725 (40.5)	3995 (40.5)	1915 (35.9)	820 (40.9)	695 (34.8)
Delta-omicron	195 475 (6.1)	194 125 (6.1)	1345 (4.6)		25 (6.6)	340 (4.3)	70 (3.9)	455 (4.6)	240 (4.5)	100 (5.0)	115 (5.8)
Omicron	1 014 855 (31.5)	1 005 610 (31.5)	9240 (31.6)		125 (32.9)	2470 (31.4)	200 (11.2)	3270 (33.1)	1880 (35.3)	600 (29.9)	695 (34.8)
**Mortality**											
Death	295 (0.0)	130 (0.0)	165 (0.6)		<10 (<2.6)	30 (0.4)	<10 (<0.6)	60 (0.6)	15 (0.3)	30 (1.5)	20 (1.0)
Hospital death:	70 (0.0)	<10 (0.0*)	65 (0.2)		<10 (<2.6)	10 (0.1)	0 (0.0)	30 (0.3)	<10 (<0.2)	15 (0.7)	<10 (<0.5)
Covid-19 or PIMS-TS related	70 (0.0)	20 (0.0)	55 (0.2)		<10 (<2.6)	<10 (<0.1)	0 (0.0)	35 (0.4)	<10 (<0.2)	<10 (<0.5)	<10 (<0.5)
Covid-19 or PIMS-TS underlying	55 (0.0)	15 (0.0)	40 (0.1)		0 (0.0)	<10 (<0.1)	0 (0.0)	30 (0.3)	<10 (<0.2)	<10 (<0.5)	0 (0.0)
Covid-19 or PIMS-TS related hospital death	40 (0.0)	0 (0.0)	40 (0.1)		<10 (<2.6)	<10 (<0.1)	0 (0.0)	25 (0.3)	<10 (<0.2)	<10 (<0.5)	<10 (<0.5)
Covid-19 or PIMS-TS underlying hospital death	30 (0.0)	0 (0.0)	30 (0.1)		0 (0.0)	<10 (<0.1)	0 (0.0)	25 (0.3)	<10 (<0.2)	<10 (<0.5)	0 (0.0)

PIMS-TS=paediatric Inflammatory multisystem syndrome with a temporal association with SARS-CoV-2.

* Counted only when test occurred 14 days before date of admission up to discharge—this window defines an admission as SARS-CoV-2-related, and it could be (but equally may not be the sole) reason for the inclusion of these participants in the applicable group.

**Fig 2:**
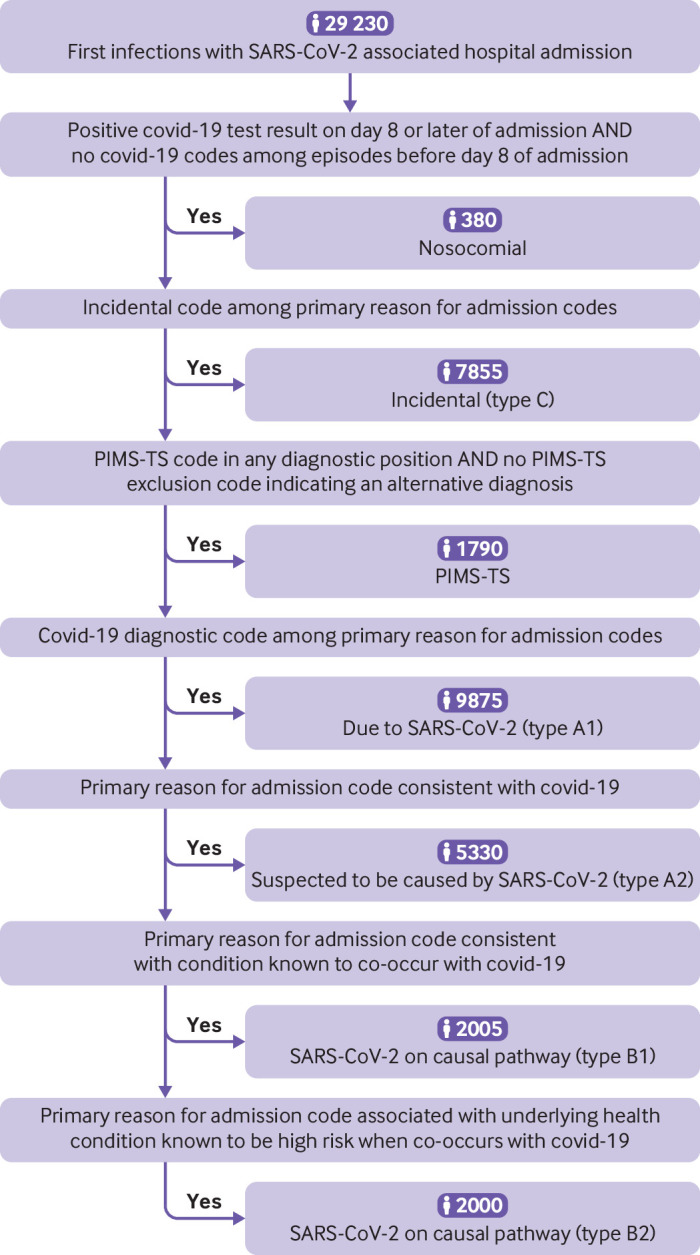
Algorithm for hierarchical classification of SARS-CoV-2 associated hospital admissions into seven subtypes. PIMS-TS=paediatric inflammatory multisystem syndrome with a temporal association with SARS-CoV-2

Of the 29 230 participants admitted to hospital, SARS-CoV-2 was deemed to have been the cause of or contributed to the admission in 21 000 (71.8%): 9875 (33.8%) of these hospital admissions were classified as type A1 (due to SARS-CoV-2 infection); 5330 (18.2%) were classified as type A2 (suspected to be due to SARS-CoV-2); 4000 (13.7%) were classified as type B (SARS-CoV-2 was a contributory factor); and 1790 (6.1%) were classified as paediatric inflammatory multisystem syndrome. Only 380 (1.3%) were classified as hospital admissions with nosocomial infection, and 7855 (26.9%) were classified as type C (a condition incidental to infection) (fig 2).

**Fig 3:**
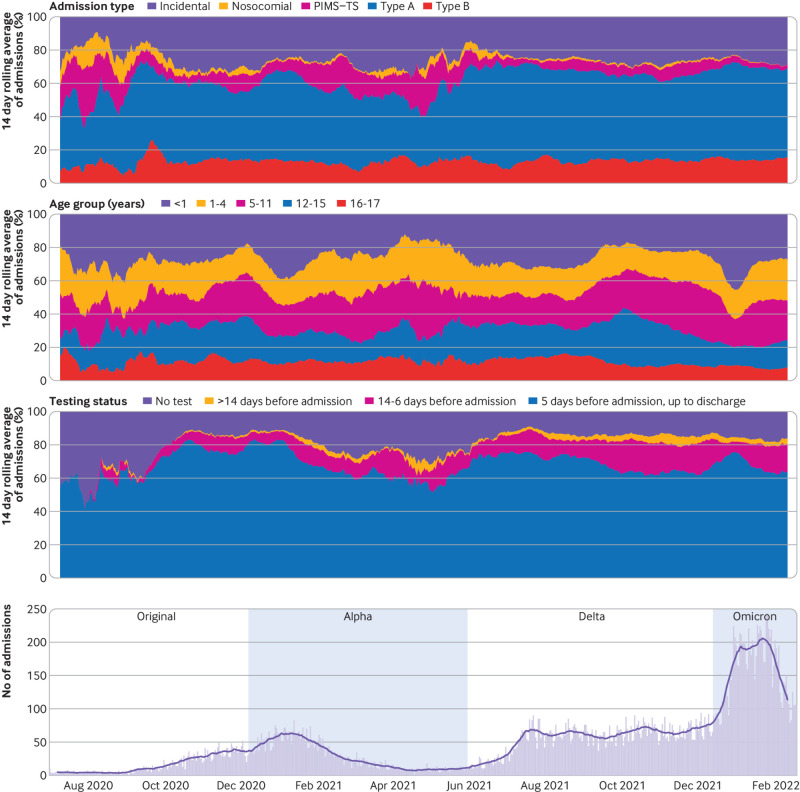
Characteristics of SARS-CoV-2 associated hospital admissions over time. Top three panels illustrate proportional changes in the 14 day rolling average of SARS-CoV-2 associated hospital admissions across three different stratifications of the cohort. First panel shows the hospital admission types. Second panel shows the participant age groups. Third panel shows the SARS-CoV-2 testing status of participants (ie, whether there was evidence of a positive test result alongside the hospital admission and the date of this test). Bottom panel shows the daily count of hospital admissions during the study period, with the black line indicating the 14 day rolling average of these counts. PIMS-TS=paediatric inflammatory multisystem syndrome with a temporal association with SARS-CoV-2

*Testing by hospital admission type*—A positive SARS-CoV-2 test result was present in 23 750 (81.3%) (type A1 8135 (82.4%), type A2 4615 (86.6%), type B 3490 (87.2%), paediatric inflammatory multisystem syndrome 130 (7.3%), nosocomial 380 (100%), and type C 7005 (89.2%)), and the remaining participants were included based on ICD-10 diagnostic codes alone. Figure 3 illustrates these stratifications over time.

*Age by hospital admission type*—Participants with type A1 admissions were the youngest (median age 1.3 (interquartile range 0.2-10.1) years), followed by participants with type A2 admissions (4.1 (1.1-11.3) years), type B admissions (6.4 (0.6-12.9) years), and paediatric inflammatory multisystem syndrome (7.6 (3.8-11.3) years). Participants with type C admissions were the oldest (10.6 (3.5-15.0) years).

*Severity of hospital admissions*—The greatest severity of SARS-CoV-2 infection was in those participants admitted to hospital with paediatric inflammatory multisystem syndrome (29.9% admitted to ICU or HDU, median stay 6 (interquartile range 4-8) days) and those admitted with nosocomial infection (27.6% admitted to ICU or HDU, median stay 93 (42-162) days). The lowest severity infections were in those with type A1 admissions (3.2% admitted to ICU or HDU, median stay 2 (1-3) days) and type A2 admissions (1.8% admitted to ICU or HDU, median stay 2 (1-2) days). Participants with type B admissions (10.1% admitted to ICU or HDU, median stay 2 (1-5) days) had medium severity infection, probably reflecting the higher rate of underlying health conditions in this group, and participants with type C admissions had lower severity symptoms (3.2% admitted to ICU or HDU, median stay 2 (1-4) days).

### Personal characteristics

Male participants (15 375 (52.6%) admitted to hospital *v* 1 593 130 (49.8%) not admitted) were overrepresented in hospital admissions compared with female participants (13 855 (47.4%) *v* 1 604 174 (50.2%)) (P<0.001). Younger children were overrepresented in hospital admissions: <1 year olds (8415 (28.8%) admitted to hospital *v* 32 940 (1.0%) not admitted) (P<0.001), whereas the opposite was found in older children—for example, 5-11 year olds (6840 (23.4%) *v* 1 454 290 (45.5%) (P<0.001). Asian or Asian British participants (4360 (15.0%) admitted to hospital *v* 270 510 (8.5%) not admitted) and black or black British participants (1860 (6.4%) *v* 85 865 (2.7%)) were overrepresented among hospital admissions, whereas the opposite was found in white participants (20 180 (69.0%) *v* 2 602 620 (81.4%)) (all P<0.001). Participants in the highest deprivation fifth were overrepresented among hospital admissions (8845 (30.3%) admitted to hospital *v* 616 440 (19.3%) not admitted) (P<0.001), whereas in participants in the lowest deprivation fifth the opposite was found (4045 (13.8%) *v* 727 760 (22.8%) (P<0.001).

### Underlying health conditions

Table 2 provides details of the pre-existing health conditions. We identified evidence of an underlying health condition flagged by the Joint Committee on Vaccination and Immunisation as contributing to clinical vulnerability, current pregnancy, and severe obesity in those older than 16 year olds^33^ in 11 085 (37.9%) of the participants admitted to hospital compared with 569 110 (17.8%) of those not admitted (P<0.001). We found evidence of a broader range of medical and developmental underlying health conditions in 15 850 (54.2%) of participants admitted to hospital compared with 892 110 (27.9%) not admitted (P<0.001). Of those participants admitted to ICU or HDU care, 1320 (77.2%) had evidence of a medical and developmental underlying health condition, and among children who died with covid-19 or paediatric inflammatory multisystem syndrome listed as a cause, a medical and developmental underlying health condition was identified in 50 of the 55 (90.9%) participants with a SARS-CoV-2 related hospital admission.

**Table 2:** Number (%) of participants with underlying health conditions for first ascertained SARS-CoV-2 infections, shown for children and adolescents not admitted to hospital, those with SARS-CoV-2 associated hospital admissions, those requiring ICU or HDU care, and those who died with covid-19 or paediatric inflammatory multisystem syndrome listed as one of the causes

Variables	First ascertained SARS-CoV-2 infections			ICU/HDU subset	Covid-19 or PIMS-TS related death
	Total	Positive SARS-CoV-2 test result only	Covid-19 associated hospital admission		
Total No of participants in group	3 226 535	3 197 305	29 230	1710	70
**Health condition linked to greater vulnerability**					
Total No with health condition	580 195 (18.0)	569 110 (17.8)	11 085 (37.9)	835 (48.8)	55 (78.6)
Health conditions:					
Cancer (excluding benign tumours)	4515 (0.1)	3495 (0.1)	1020 (3.5)	40 (2.3)	<10 (<14.3)
Blood disorders and immune deficiencies	11 765 (0.4)	10 665 (0.3)	1095 (3.7)	70 (4.1)	<10 (<14.3)
Endocrine conditions	20 985 (0.7)	19 570 (0.6)	1420 (4.9)	125 (7.3)	15 (21.4)
Severe neurological and developmental conditions	119 910 (3.7)	117 140 (3.7)	2770 (9.5)	265 (15.5)	35 (50.0)
Hypertension, cardiac valve disorders, and cardiomyopathy	10 765 (0.3)	9625 (0.3)	1140 (3.9)	195 (11.4)	10 (14.3)
Severe respiratory diseases	399 610 (12.4)	394 655 (12.3)	4950 (16.9)	260 (15.2)	20 (28.6)
Digestive, liver, and renal diseases	6605 (0.2)	5810 (0.2)	790 (2.7)	35 (2.0)	<10 (<14.3)
Arthritis and connective tissue diseases	8870 (0.3)	8490 (0.3)	380 (1.3)	20 (1.2)	0 (0.0)
Congenital syndromes and anomalies	68 875 (2.1)	65 930 (2.1)	2950 (10.1)	375 (21.9)	35 (50.0)
Obesity (age >16 years)	6245 (0.2)	6020 (0.2)	220 (0.8)	<10 (<0.6)	0 (0.0)
Pregnancy	4425 (0.1)	4110 (0.1)	315 (1.1)	0 (0.0)	0 (0.0)
**Medical and developmental underlying health conditions identified by the study team**					
Total No with health condition	907 960 (28.1)	892 110 (27.9)	15 850 (54.2)	1315 (76.9)	65 (92.9)
Health condition:					
Cancer and neoplasms (excluding benign tumours)	5575 (0.2)	4465 (0.1)	1110 (3.8)	55 (3.2)	<10 (<14.3)
Blood disorders and immune deficiencies	23 585 (0.7)	21 225 (0.7)	2360 (8.1)	170 (9.9)	<10 (<14.3)
Endocrine conditions	101 105 (3.1)	97 610 (3.1)	3495 (12.0)	320 (18.7)	30 (42.9)
Neurological and developmental conditions	256 000 (7.9)	250 045 (7.8)	5955 (20.4)	535 (31.3)	50 (71.4)
Respiratory conditions	489 645 (15.2)	482 900 (15.1)	6745 (23.1)	400 (23.4)	40 (57.1)
Hypertension and heart disease (congenital and acquired)	325 870 (10.1)	320 770 (10.0)	5100 (17.4)	630 (36.8)	35 (50.0)
Digestive and liver conditions	49 695 (1.5)	47 705 (1.5)	1985 (6.8)	145 (8.5)	10 (14.3)
Muscle, skin, and arthritis	55 940 (1.7)	53 835 (1.7)	2105 (7.2)	220 (12.9)	30 (42.9)
Renal and genitourinary conditions	88 410 (2.7)	86 425 (2.7)	1985 (6.8)	140 (8.2)	15 (21.4)
Prematurity and low birth weight	31 595 (1.0)	29 155 (0.9)	2440 (8.3)	390 (22.8)	10 (14.3)
Evidence of obesity	83 405 (2.6)	81 980 (2.6)	1425 (4.9)	80 (4.7)	<10 (<14.3)
Evidence of recent or current pregnancy	4425 (0.1)	4110 (0.1)	315 (1.1)	0 (0.0)	0 (0.0)

HDU=high dependency unit; ICU=intensive care unit; PIMS-TS=paediatric Inflammatory multisystem syndrome with a temporal association with SARS-CoV-2.

Information on participants’ weight was poor (available in the previous two years for 48.1% of those admitted to hospital and 20.5% of those not admitted). Based on available information, there was evidence of obesity in 1425 (4.9%) of the participants admitted to hospital, and a lower proportion 81 980 (2.6%) among those not admitted (P<0.001). Pregnancy was reported in 315 (1.1%) of participants admitted to hospital and a lower rate 4110 (0.1%) among those not admitted (P<0.001).

### Vaccination in eligible participants

In adolescents older than 12 years with a first ascertained SARS-CoV-2 infection after 19 July 2021, when the use of the Pfizer-BioNTech covid-19 vaccine was approved for use in vulnerable 12-15 year olds, and in all adolescents older than 16 years, the proportion vaccinated increased over time (see supplementary figure B). When we explored vaccination (defined as one dose or more, see supplementary table D), we found that 782 490 (69.4%) were unvaccinated among the 1 127 735 participants not admitted to hospital and 4050 (74.7%) were unvaccinated among the 5420 who were admitted, with higher unvaccinated proportions in those who required ICU or HDU care: 160 of 180 (88.9%) (P<0.001). Overall, 232 425 (20.5%) of participants with a first ascertained infection had evidence of a clinical condition that was linked to greater vulnerability for severe disease with SARS-CoV-2.^33^ In these higher risk participants, the unvaccinated proportions followed a similar trend: 151 860 (66.1%) participants who were not admitted to hospital, 2010 (70.9%) admitted to hospital, and 95 (86.4%) admitted to ICU or HDU care (P<0.001).

### Trends including SARS-CoV-2 associated hospital admission rate 

The rate of first ascertained SARS-CoV-2 associated hospital admissions was observed to increase between the original and alpha waves (rate 1.9% to 2.1%), and then decline in the delta (0.7%) and omicron (0.9%) waves (table 3, also see supplementary figure C). The number of children and adolescents admitted to hospital during the original and alpha waves (2445 and 3300, respectively) was substantially lower than those admitted to hospital during the delta and omicron waves (11 185 and 9240, respectively); we only captured infections from the omicron era for seven weeks plus the six weeks of follow-up for admissions and did not capture infections in periods where variants overlapped (fig 1 and table 3). The proportions of hospital admissions by type did not vary much by wave, except for paediatric inflammatory multisystem syndrome, which decreased over time (original 230 (9.4%), alpha 425 (12.9%), delta 725 (6.5%), omicron 200 (2.2%)); as did the proportion of participants who required ICU or HDU care (original 170 (7.0%), alpha 325 (9.8%), delta 675 (6.0%), omicron 355 (3.6%)). The rate of hospital admissions also decreased over time for those with paediatric inflammatory multisystem syndrome and for those requiring ICU or HDU care. 

**Table 3:** Number (percentage) of first ascertained SARS-CoV-2 associated hospital admissions by type for each variant wave

	Variant period			
	Original: 1 Jul 2020-5 Dec 2020	Alpha: 3 Jan 2021-1 May 2021	Delta: 30 May 2021-11 Dec 2021	Omicron: 26 Dec 2021-17 Feb 2022
No of first ascertained infections	127 210	158 960	1 612 630	1 014 855
**Covid-19 hospital related admission types**				
All types of hospital admissions combined	2445 (1.9)	3300 (2.1)	11 185 (0.7)	9240 (0.9)
Specific types:				
Type A (due to or suspected to be due to SARS-CoV-2)	1120 (0.9)	1525 (1.0)	5910 (0.4)	5150 (0.5)
Type B (SARS-CoV-2 on causal pathway)	335 (0.3)	395 (0.2)	1510 (0.1)	1295 (0.1)
Paediatric inflammatory multisystem syndrome	230 (0.2)	425 (0.3)	725 (0.0)	200 (0.0)
ICU or HDU care	170 (0.1)	325 (0.2)	675 (0.0)	335 (0.0)

HDU=high dependency unit; ICU=intensive care unit.

Data for the periods when variants overlapped are not shown. The rate of SARS-CoV-2 associated hospital admission during each wave is calculated as the percentage of first infections starting in each period.

## Discussion

We describe first ascertained SARS-CoV-2 infections and SARS-CoV-2 associated hospital admissions for 3.2 million children and adolescents, among the estimated population of 12 million in England,^12^ during the covid-19 pandemic when the community testing programme was active. To contextualise this, we displayed notable events, including changes in testing policies (fig 1), alongside trends in infections. SARS-CoV-2 either caused or was a contributory factor in most (71.8%) SARS-CoV-2 associated hospital admissions in children and adolescents. The median stay was two days, implying rapid recovery for most; however, 5.9% of hospital admissions involved ICU or HDU care, thus representing more serious illness. Nonetheless, our findings of higher hospital admission rates in boys,^45^ hospital admission, ICU or HDU care, death with underlying health condition,^11^ ^19^ ^23^ ^30^ hospital admission, ICU or HDU care in people from ethnic minority groups 11 19 23 30 45 and more deprived backgrounds,^30^ ^45^ are entirely consistent with the findings of a range of previous, smaller studies of differing designs, thus supporting the validity of our study.

### Study findings in context

#### Reasons for hospital admission

We found a higher number of SARS-CoV-2 associated hospital admissions than the number of covid-19 admissions reported by NHS England in the same period (29 230 *v* 21 476).^7^ An explanation for this is that our more inclusive methods captured a wider range of hospital admissions associated with a SARS-CoV-2 infection than the methods used by NHS England; both approaches capture incidental hospital admissions. The inclusion of not only hospital admissions due to SARS-CoV-2 infection but also admissions where this infection was strongly suspected or part of a more complex combination of causal factors, was motivated by clinician and patient and public involvement feedback. Our panel of consultant paediatricians highlighted scenarios without a classic covid-19 presentation, where SARS-CoV-2 is likely to be implicated (some reported in case series^30^ ^46^ ^47^ ^48^ ^49^) or to contribute to hospital admission with mild SARS-CoV-2 infection, owing to concern for complications in the context of underlying health conditions.^30^ ^50^ ^51^ Patient and public involvement feedback about children and adolescents who experienced hospital admissions with SARS-CoV-2 as a contributor or incidental finding, highlighted the psychosocial burdens of isolation and the need to cancel or delay investigations or procedures, with a potential detriment to health.

The degree to which SARS-CoV-2 leads to hospital admission has been widely debated, in part because this is an emerging disease that clinicians and researchers are still learning about. We found a higher proportion of incidental admissions than did the International Severe Acute Respiratory and emerging Infection Consortium (ISARIC) study (26.9% *v* 20.6%),^19^ and in contrast with NHS England data, which is dominated by adults,^7^ we found no increase in numbers of patients admitted with incidental infection during the omicron wave. We may have designated a wider range of diagnostic codes as being incidental compared with ISARIC, based on codes observed in the electronic health record. Paediatric studies from the first pandemic wave in the UK noted the rarity of severe disease in children and young people,^10^ ^11^ ^22^ ^23^ which might mean that paediatric experience of this condition was initially restricted but has evolved over time. The low rate of nosocomial SARS-CoV-2 infection in children and adolescents (1.3%) might reflect the shorter stays and smaller number of infected patients in children’s wards compared with what is observed in adult wards.^7^

#### Severe hospital admissions

Between March 2020 and January 2022 for the UK and Republic of Ireland, the Paediatric Intensive Care Audit network (PICANet) reported paediatric ICU (PICU) admissions in 714 people younger than 17 years with SARS-CoV-2 and 818 with paediatric inflammatory multisystem syndrome (total 1532).^52^ In our slightly later study period (incorporating less data from the original variant era and more from the omicron era) for England we reported 1710 hospital admissions involving critical care, of which 535 were admissions with paediatric inflammatory multisystem syndrome, leaving 1175 young people admitted to ICU or HDU who did not have paediatric inflammatory multisystem syndrome (14.9% of these were Type C incidental). Our study captured HDU care outside a designated PICU, and it had an older upper age limit of 18 years; therefore, our numbers are compatible with those of PICANet. For context, the 1105 SARS-CoV-2 associated ICU or HDU admissions for the last year of our study can be considered against the pre-pandemic (2016-19) annual mean numbers from PICANet for more familiar conditions: 1820 for bronchiolitis (mainly in infants), 419 for trauma, and 399 for asthma exaccerbations.^29^

The Joint Committee for Vaccination and Immunisation identified hospital admission involving critical care or paediatric inflammatory multisystem syndrome as severe outcomes and we concur with this.^43^ ^44^ Nonetheless, our ascertainment of children and adolescents with paediatric inflammatory multisystem syndrome was complicated as this new syndrome was described in May 2020,^22^ hence the codes used for paediatric inflammatory multisystem syndrome (M30.3 and R65, see box 1) had other meanings before the pandemic. Of 1790 children and adolescents with paediatric inflammatory multisystem syndrome in our study, 1085 were assigned to the code U07.5 (code used since November 2020). Of those hospital admissions remaining, 20 involved both M30.3 and R65; 600 involved only M30.3; and 85 involved only R65; with no evidence of an alternative cause, as determined by exclusion codes. In 2019, when the rates of acute hospital admissions in children and adolescents were higher than during the pandemic, after excluding those with an alternative cause, five were coded as R65 (systemic inflammation) and 305 as M30.3 (Kawasaki disease), of which 10 involved ICU or HDU care. While personal characteristics and test positivity rates (27.0% tested positive 42 days before hospital admission) indicate similarities between the participants with paediatric inflammatory multisystem syndrome in our cohort and those in other studies, supporting our case ascertainment,^20^ ^21^ ^22^ we acknowledge that a small proportion of cases defined by M30.3, especially among ward level hospital admissions with paediatric inflammatory multisystem syndrome, could have had Kawasaki disease. Moreover, our observed reduction in number of young people admitted to hospital with paediatric inflammatory multisystem syndrome over time is consistent with reports from Denmark^53^ and the United States,^54^ and from multicentre data from 2022^55^: the reasons are unknown but may relate to increasing rates of previous exposure to SARS-CoV-2 and to covid-19 vaccination.

#### High risk groups

The underlying health conditions most notably affecting children and adolescents admitted to hospital more than those not admitted, were respiratory (23.1% of those admitted to hospital), particularly asthma, which is plausible given the respiratory symptoms of covid-19, and neurological (20.4% of those admitted to hospital), such as autistic spectrum disorders and developmental delays, which hinder social distancing and the ability to describe symptoms and hence access to care. Although children younger than 5 years, who are ineligible for vaccination in England, were less likely to access testing (for example, they were never tested in a school setting) and were under-represented among those participants with ascertained SARS-CoV-2 infections; consistent with the ISARIC study,^19^ ^23^ they were over-represented among participants admitted to hospital. As well as the possibility of more severe disease in young children, another explanatory factor is that clinicians treat babies with more caution because they are more difficult to assess when acutely unwell and more likely to require a period of observation or support with fluid intake than older children. It is concerning that as well as suggesting greater disease severity, our data may indicate that black children and adolescents and those from high deprivation backgrounds, were least likely to access testing. Black or black British ethnicity accounts for 3.3% within the wider English population,^56^ and 24.0% of children and adolescents live in the most deprived areas.^57^ The respective proportions of children and adolescents admitted to hospital were higher than expected (6.4% and 30.3%), whereas for infected children and adolescents not admitted to hospital, these proportions were lower than expected (2.7% and 19.3%).

#### Variant waves

A population based study from the UK found that in contrast with adults, children and adolescents had a similar risk of hospital admission with delta and omicron to previous variants.^58^ When interpreting the decrease in hospital admission rate while infections and hospital admissions were higher with delta and omicron, it should be remembered that although the clinical criteria for hospital admission in unwell children remained similar over the study period, strong evidence suggests that during the early phase of the pandemic (original and alpha eras) much less healthcare was accessed^59^ ^60^ ^61^ (children and adolescents were less likely to be tested and present to hospital). Conversely, use of testing was greater during the delta and omicron eras, in particular the use of lateral flow tests in secondary schools from March 2021 was associated with an increase in ascertainment of milder infections. Moreover, our focus was on first infections, not reinfections, which are more likely over time. Finally, the omicron variant may be relatively less severe than previous variants.

### Strengths and limitations of this study

Our study has the advantage of being based on a linked dataset representing children and adolescents in England and therefore is on a national scale. Ascertainment of infections in children and adolescents who were not admitted to hospital, depended only on test results and as such, will have been influenced by differential availability and uptake of testing. A subset of children and adolescents who had a SARS-CoV-2 associated hospital admission, were included on the basis of a clinical illness and no positive test result. Given this limitation, for comparison, we present rates of SARS-CoV-2 associated hospital admission for children and adolescents based on only those first ascertained infections with a positive SARS-CoV-2 test result (see supplementary table E); all values were slightly lower than those in table 3. Of children and adolescents with a SARS-CoV-2 associated hospital admission, 18.7% were ascertained solely by codes, that will have been informed by clinical assessment, which is subjective and influenced by experience. A motive for including these participants is that children and adolescents might fail to receive a positive test result in the UK Health Security Agency’s Second Generation Surveillance System and yet be judged by clinicians as infected with SARS-CoV-2 from in-person evaluation of the illness, informed by the patient’s history of contacts and lateral flow test result. A weakness of case ascertainment using clinical codes, is that coding quality is determined by the availability of adequate diagnostic codes. During the emergence of a new illness, as well as inherent limitations in data quality and discrimination, coding quality could have been affected by a learning curve (supplementary table F shows the percentage prevalence of each covid-19 code for the hospital admission types). Although we wanted to identify conditions that were linked with clinical vulnerability to severe disease, our match with the Green Book^33^ was imperfect for conditions with a range of severity. Our methods will have led to over ascertainment and inclusion of low severity underlying health conditions thus leading to high estimates. Because we focused on children and adolescents admitted for acute illness, 6410 were excluded where the only evidence was a non-primary reason for admission with a history of covid-19 (U07.3) or a post-covid-19 condition (U07.4), as we were concerned that this pattern of coding might represent recording of patient history. Focusing on acute conditions meant that chronic post-covid-19 health conditions were not considered.

### Conclusions

Access to key relevant datasets via the CVD-COVID-UK/COVID-IMPACT consortium^14^ enabled the creation of a linked cohort, and iterative exploratory analysis, involving close collaboration between data scientists and clinicians. We found that for most (21 000 of 29 230 (71.8%)) of the children and adolescents with SARS-CoV-2 associated hospital admission, this was due to SARS-CoV-2 infection or SARS-CoV-2 was a contributory factor. Although most of these participants had short hospital stays, 1710 (5.9%) received critical care, and a further 1255 (4.3%) participants were admitted with paediatric inflammatory multisystem syndrome but did not require critical care. Children and adolescents from ethnic minority groups and more deprived backgrounds were disproportionately represented by these more severe outcomes of SARS-Cov-2 infection. The proportion of children and adolescents admitted to hospital with severe disease decreased during the omicron variant era, largely driven by a reduction in paediatric inflammatory multisystem syndrome related hospital admissions. Our initial exploratory analyses reinforce the need for targeted public health action (eg, vaccination uptake associated interventions) to protect children and adolescents from ethnic minority groups and deprived backgrounds who seem to be disproportionately at risk of SARS-CoV-2 infection and hospital admission.

**What is already known on this topic**

Although hospital admission of children and adolescents with SARS-CoV-2 is rare, infection rates have been high and those with underlying health conditions are at increased risk of severe outcomes
Covid-19 vaccination rates are low in children and adolescents
Between autumn 2021 and spring 2022 children and adolescents experienced high rates of SARS-CoV-2 infection, but the implications of this for hospital admissions were unclear

**What this study adds**

In children and adolescents admitted to hospital with a first ascertained SARS-CoV-2 infection, 21 000 of 29 230 (71.8%) were admitted due to the virus or with the virus as a contributory factor
Children and adolescents from ethnic minority groups and more deprived backgrounds were disproportionately represented among participants admitted to hospital, the 1710 of 29 230 (5.9%) who were admitted requiring critical care, and the further 1255 (4.3%) who were admitted with paediatric inflammatory multisystem syndrome but did not receive critical care
The proportion of children and adolescents with severe disease decreased during the omicron variant era, and falling numbers of participants with paediatric inflammatory multisystem syndrome contributed to this decline

## Data Availability

This analysis was performed according to a prespecified analysis plan published on GitHub. Supplementary table A provides all the ICD-10 codes used to define admission types and additional technical details, which are publicly available with analytical code on GitHub https://github.com/BHFDSC/CCU029_01.^62^ The data used in this study are safeguarded in NHS England’s Trusted Research Environment (TRE) for England, but as restrictions apply the data are not publicly available (https://digital.nhs.uk/coronavirus/coronavirus-data-services-updates/trusted-research-environment-service-for-england). The CVD-COVID-UK/COVID-IMPACT programme, led by the British Heart Foundation Data Science Centre, (https://www.hdruk.ac.uk/helping-with-health-data/bhf-data-science-centre/) received approval to access data in NHS England’s TRE for England from the Independent Group Advising on the Release of Data (IGARD) (https://digital.nhs.uk/about-nhs-digital/corporate-information-and-documents/independent-group-advising-on-the-release-of-data) via an application made in the Data Access Request Service (DARS) Online system (ref DARS-NIC-381078-Y9C5K) (https://digital.nhs.uk/services/data-access-request-service-dars/dars-products-and-services). The CVD-COVID-UK/COVID-IMPACT Approvals and Oversight Board (https://www.hdruk.ac.uk/projects/cvd-covid-uk-project/) subsequently granted approval of this project to access the data within NHS England’s TRE for England. The deidentified data used in this study were made available to accredited researchers only. Those wishing to gain access to the data should contact bhfdsc@hdruk.ac.uk in the first instance.

## Web extra

Extra material supplied by authors

## Notes

Contributors: HW, CT, BAM, DS, AGI, DG, CP, SD, SV, and KB designed the study. HW primarily undertook data analysis, with input from CT, BAM, DS, CP, DG, SD, SV, and KB. KB and HKK constructed the clinical coding maps. HKK, PR, PDP, MJ, NP, and KB provided clinical contributions to the design and interpretation of data. HW and AL produced the original figures. HW and KB wrote the first draft of the paper. All authors read and contributed to the final version of the manuscript. KB is the guarantor. The corresponding author attests that all listed authors meet authorship criteria and that no authors meeting the criteria have been omitted.

The lead author (KB) affirms that the manuscript is an honest, accurate, and transparent account of the study being reported; that no important aspects of the study have been omitted; and that any discrepancies from the study as originally planned (and, if relevant, registered) have been explained.

Dissemination to participants and related patient and public communities: Parents and parent groups kindly provided informal comments on experiences across the range of admission types, which informed our research. We will share a summary of the research with representatives of the parent organisations Parents Utd, SafeEdForAll, LongCovidKids, OneVoice, ShieldUs, Clinically Vulnerable Families UK, and the Hazards Campaign, as well sharing the work through our institutions.

Provenance and peer review: Not commissioned; externally peer reviewed.
