# Supplementary material for: Hospital admissions linked to SARS-CoV-2 infection in children and adolescents: cohort study of 3.2 million first ascertained infections in England
Source: BMJ. 2023 Jul 5;382:e073639. doi: 10.1136/bmj-2022-073639 (PMC10318942; doi:10.1136/bmj-2022-073639)
Supplement: Supplementary file 1 — Supplementary information: Additional tables A-F and additional figures A-C [file wilh073639.ww.pdf]

Supplementary Materials

Hospital admissions linked to SARS-CoV-2 infection: a cohort study of 3.2 million first ascertained infections affecting children resident in England

Table A: Information on admission type codes for stated groups of SARS-CoV-2 associated hospital admissions

Type of Admission	Criteria for Inclusion<br>ICD-10 Codes presented in the format provided by the World Health Organisation 2019:<br>https://icd.who.int/browse10/2019/en<br>Where we present a code as a single letter, then we include the whole chapter (eg: C); where we present a code as a letter with a single number (eg: C0) we include all codes starting with this letter number combination; where we present a code as a letter with two numbers (eg: C00) we include all codes starting with this letter number combination; and where we present a code with a letter and three numbers with a decimal point (eg: C00.0) we include only this specific code.
Hospitalisation associated with Nosocomial SARS-CoV-2 infection	A first associated Positive SARS-CoV-2 test between day 8 of hospitalisation and hospital discharge and / or first presence of Covid codes U07.1, U07.2 (and U07.3, U07.4 **only** in primary position) amongst causes for hospitalisation from episodes starting after day 8 of the admission.
Type C (Incidental) (Hospitalisation associated with incidental SARS-CoV-2 infection)	Mental health, eye conditions, dental conditions, injuries, trauma, assault, self-harm, poisoning, surgical problems such as those affecting hernia, bowel or testis, certain pregnancy related conditions, neonatal disorders with clear non covid related aetiology, infections with no known link to SARS-COV-2, skin disorders, health care process codes excepting isolation cubicles.<br>The above are represented by the following codes / chapters in a primary diagnostic position:<br>A00, A01, A02, A03, A04, A05, A06, A07, A080, A081, A082, A15, A16, A17, A18, A19, A2, A3, A5, A6, B00, B01, B02, B03, B04, B05, B06, B07, B08, B1, B26, B3, B4, B5, B6, B7, B8, D1, D2, D30, D31, D32 ,D33, D34, D35, D36, D50, D51, D52, D53, D63.8, D64.9, D68.9, D69, D73, E0, E15, E16, E2, E3, E5, E87, F0, F1, F2, F3, F4, F5, F6, F70, F71, F80, F81, F82, F9, G0, G25.0, G25.1, G25.2, G25.3, G25.4, G25.5, G25.6, G25.8, G43, G44, G5, G6, G9, H, I01, I02, I33, I38, I39, I6, I7, I8, J6, J95, K0, K1, K2, K3, K4, K55, K56, K58, K59, K6, K77, K8, K91, K92, K93, L0, L2, L3, L4, L5, L6, L72, L73, L8, L9, M00, M01,M02, M03, M1, M2, M4, M5, M6, M7, M8, M9, N10, N14, N16, N2, N3, N39, N4, N5, N6, N7, N8, N9, O, P00, P01, P02, P03, P04, P08, P1, P20, P21, P24, P26, P3, P50, P51, P52, P53, P54, P55, P56, P57, P58, P59, P6, P70, P71, P72, P75, P76, P77, P78, P8, P90, P91, P94, P95, P96, Q1, Q38, Q5, Q66, Q68, Q69, Q70, Q71, Q72, Q73, Q74, Q82, Q84, Q86, Q95, Q96, Q97, Q98, Q99, R04, R01, R03, R09, R12, R14, R15, R19, R20, R22, R23, R25, R26, R27, R29.0, R29.1, R29.2, R29.4, R30, R31, R32, R33, R35, R39, R44, R45, R46, R47, R60, R61, R63.1, R63.2, R68.0, R76.8, R80, R81, R82, R89, R90.0, R93, U07.0, U07.6, U82, U83, S, T, V, W, X, Y, Z<br>Excluding these codes representing different subgroups in primary positions, and the three PIMS-TS related codes (U07.5, M30.3, R65) in non-primary positions:<br>B30.9, B33.8, B34, H66.9, I88.0, K29.7, K44, K85.9, Y4, Y5, Z03.8, Z03.9, U07.5, M30.3, R65
PIMS-TS (Hospitalisation with Paediatric Inflammatory Multisystem Syndrome with a temporal association with SARS-COV-2)	PIMS-TS code in any diagnostic position (U07.5, M30.3, R65), and in the case of M30.3 and R65, none of the following exclude codes that indicate a diagnosis of sepsis or bacterial diseases may be more likely than PIMS-TS:<br>A00, A01, A02, A03, A04, A05, A06, A07, A08.0, A08.1, A08.2, A17, A18, A19, A2, A3, A4, A5, A6, A7, A80, A81.0, A81.1, A81.2, A82, A83, A84, A85, A87.0, A87.1, A88.0, A88.1, A9, B01, B02, B03, B04, B05, B06, B07, B08, B15, B16, B17, B18, B2, B30.0, B30.1, B30.3, B33.0, B33.1, B33.3, B33.4, B34.0, B34.1, B34.3, B34.4, B4, B5, B6, B7, B8, B90, B91, B92, B94, B95, B96, B97.0, B97.1, B97.3, B97.4, B97.5, B97.6, B97.7, B98, C, D37, D38, D4, D5, D60, D61, D62, D63, D65, D66, D67, D8, E88.3, G00, G01, G02, G03.0, G03.1, G03.2, G04.1, G04.2, G05, G06, G07, G08, I0, I31, 132, I35, I36, I37, I38, I39, Q2, J02.0, J03.0, J09, J10, J11, J12.0, J12.1, J12.2, J12.3, J13, J14, J15, J16.0, J17, J20.0, J20.1, J20.2, J20.3, J20.4, J20.5, J20.6, J20.7, J21.0, J21.1, J21.8, J36, J39.0, J39.1, J69, J85, J86, J94, J95, L0, M0, M01, M03, O, P0, P1, P21, P23, P24, P26, P35.0, P35.1, P35.2, P35.4, P35.4, P36, P37, P50, P51, P54, P75, P76, P77, P78.0, P78.1, P78.2, P78.3, P96.0, R02, R57.2, S, T, V, Z95.8, Z98.2, Z94.8
Type A1 (Hospitalisation caused by SARS-CoV-2 infection)	Not allocated to the types in the rows above, and one of the Covid codes in a primary diagnostic position:<br>U07.1, U07.2, U07.3, U07.4

Type A2 (Hospitalisation suspected to be caused by SARS-CoV-2 infection)	Not allocated to the types in the rows above, Covid Code in a non-primary (U07.1 or U07.2 **only**) position and/or a Positive Test, as well as codes capturing paediatric symptoms of SARS-CoV-2 infection as the primary reason for admission.<br>These symptoms included: enteritis of unknown cause, unspecified sepsis /viral conjunctivitis, /viral diseases or infections/diarrhoea/infection, coronavirus unspecified, infection associated HLH, volume depletion, shock, otitis media unspecified, coronary artery aneurysm, pulmonary embolism, tachycardias, myocarditis, mesenteric lymphadenitis unspecified, hypotension, unspecified nasopharyngitis/tonsilitis,/pharyngitis, laryngitis/pneumonia/tonsilitis/croup/bronchiolitis/bronchitis, ARDS, pneumothorax, respiratory failure, gastritis unspecified, pain in joints, unspecified acute renal failure, neonatal respiratory issues with air leak, neonatal poor feeding, cough, wheeze, sore throat, abdominal pain, vomiting, unspecific rash, abnormal movements, somnolence, disorientation, dizziness, anosmia, fever, headache, myalgia, malaise, syncope, febrile convulsion, shock, lymphadenopathy, feeding difficulties, unknown cause, abnormal respiratory exam, SARS unspecified .<br>A08.4, A08.3, A08.5, A09.0, A09.9, A41.9, B30.9, B33.8, B34.8, B34.9, B97.2, B99, D76.2, E86, G25.9, H66.9, I25.4, I26, I40, I41, I42, I46, I47, I88.0, I95, J00, J01, J02.9, J03.9, J04, J05, J06, J18, J22, J40, J80, J81, J93, J96, J98, K29.7, M25.5, N17.9, P25, P92, R00, R05, R06, R07, R09.8, R10, R11, R13, R21, R29.8,R34, R40, R41, R42, R43, R50, R51, R52, R53, R55, R56, R57, R59, R63.0, R63.3, R63.4, R63.6, R63.8, R68.1, R69, R84.5, U04.9<br><br>Exclude specific infections that might be alternative causes (primary or non-primary code position):<br>A01, A02, A03, A04, A05, A06, A06, A07, A08.0, A08.1, A08.2, B25, B27, J0.20, J0.30, J09, J10, J11, J12, J13, J14, J15, J16, J17, J20.0, J20.1, J20.2, J20.3, J20.4, J20.5, J20.6, J20.7, J21.0, J21.1
Type B1 (Hospitalisation with SARS-CoV-2 as a contributor alongside other acute conditions)	Not allocated to the types in the rows above, Covid Code in a non-primary position and/or a Positive Test, and one of the following diagnoses as a reason for admission:<br>RSV, adenovirus, staphylococcal pneumonia, streptococcal pneumonia. Conditions that have been linked to SARS-COV-2 infection appendicitis, type one diabetes, status epilepticus or febrile seizures. Treatment codes such as isolation in cubicle.<br>A01, A02, A03, A04, A05, A06, A06, A07, A08.0, A08.1, A08.2, A40, A41, A48.3, A49, A81.8, A85.8, A86, A87.9, A87.0, A87.8, A89, B09, B25, B27, B34, B95, B96, B97, E10, E87.8, G41, I30, I32, J02, J03, J09, J10, J11, J12, J13, J14, J15, J16, J17, J20, J21, J3, J85, J86, J90, K85.9, M30.3, P22, P23, P28, P29, P74, R17, R65, R70, R71, R72, R73, R74, R79, U07.5, Z03.8, Z03.9
Type B2 (Hospitalisation with SARS-CoV-2 as a contributor alongside an underlying health condition)	Not allocated to the types in the rows above, Covid Code in a non-primary position and/or a Positive Test, and one of the following diagnoses as the primary reason for admission:<br>Conditions treated with immune suppression, cancer, neurodevelopmental conditions including those that may affect breathing, endocrine disorders, neonatal conditions such as jaundice or poor feeding, respiratory diseases such as asthma and cystic fibrosis, blood disorders that can affect breathing or cause immune suppression, congenital anomalies that affect the whole body, may impair the heart or circulation or immunity or endocrine function.<br>B20, B21, B22, B24, D3, C, D0, D37, D38, D39, D4, D55, D56, D57, D58, D59, D60, D61, D64.0, D64.4, D66, D67, D68.0, D68.1, D68.2, D70, D71, D72.0, D73.0, D73.1, D76.1, D76.3, D8, E00, E10, E11, E12, E13, E14, E20, E21, E22, E23, E24, E25, E26, E27, E4, E6, E7, E80, E83, E84, E85, E88, E89, F72, F73, F78, F79, F83, F84, F88, F89, G1, G20, G21, G22, G23, G24, G3, G40, G45,G46, G47, G7, G8, G91, G94, I05, I06, I07, I08, I09, I10, I11, I12, I13, I15, I27, I28, I31, I34, I35, I36, I37, I42, I43, I44, I45, I47, I48, I49, I50, I51, I52, J38.0, J38.6, J41, J42, J43, J44, J45, J46, J47, J82, J84, J98.4, J99, K44, K50, K51, K52, K71, K72, K73, K74, K75, K76, K90, L1, L85, L93, L94, M05, M06, M07, M08, M09, M30.0, M30.1, M30.2, M31, M32.1, M32.8, M32.9, M33, M34, M35, M41, M42, M43, N0, N11, N12, N13, N15, N18, N19, P05, P07, P27, Q0, Q2, Q30, Q31, Q32, Q33, Q34, Q35, Q36, Q37, Q39, Q4, Q60, Q61, Q62, Q63, Q64, Q65, Q67, Q75, Q76, Q77, Q78, Q79, Q80, Q81, Q85, Q87, Q89, Q90, Q91, Q92, Q93, R16, R62, R56.8, R94

Table B: ICD-10 codes defining conditions associated with clinical vulnerability and other medical and developmental health conditions linked to SARS-CoV-2 associated hospitalisation

ICD-10 match to conditions specifically lagged by JCVI as leading to greater vulnerability to severe disease with SARS-CoV-2<br>https://www.gov.uk/government/publications/covid-19-the-green-book-chapter-14a	ICD-10 Codes presented in the format provided by the World Health Organisation 2019: https://icd.who.int/browse10/2019/en<br>Where we present a code as a single letter, then we include the whole chapter (eg: C); where we present a code as a letter with a single number (eg: C0) we include all codes starting with this letter number combination; where we present a code as a letter with two numbers (eg: C00) we include all codes starting with this letter number combination; and where we present a code with a letter and three numbers with a decimal point (eg: C00.0) we include only this specific code.
Cancer excluding benign tumours, (only considered within 5 years prior to appearance in cohort)	C, D0
Blood Disorders and Immune Deficiencies	B20, B21, B22, B24, D56, D57, D58, D61, D71, D72.0, D73.0, D73.1, D8
Endocrine Conditions	E10, E11, E12, E13, E14, E22, E23, E24, E25, E26, E27, E70, E71, E72, E74, E75, E76, E79, E84
Severe Neurological and Developmental Conditions	F72, F73, F84, G1, G20, G21, G22, G23, G24, G26, G3, G40, G45, G7, G8
Hypertension, Cardiac Valves and Cardiomyopathy	I10, I11, I12, I13, I15, I27, I34, I35, I36, I37, I42, I43
Severe Respiratory Diseases	B27 (only considered in the prior 5 years), J38.0, J38.6, J44, J45, J46, J47, J82, J84, J99
Digestive, Liver and Renal Diseases	K44, K50, K51, K71, K72.1, K74.0, K74.1, K74.4, K74.5, K75, N03, N04, N18.3, N18.4, N18.5
Arthritis and Connective Tissue Diseases	M05, M06, M07, M08, M09, M30.0, M30.1, M30.2, M31, M32.1, M34, M35
Certain Congenital Syndromes and Anomalies affecting heart lungs kidneys liver or multi-system	Q0, Q20, Q21.0, Q21.2, Q21.3, Q21.4, Q21.8, Q22, Q23, Q24.2, Q24.4, Q24.5, Q24.6, Q25, Q26, Q31, Q32, Q33, Q34, Q39, Q44.2, Q60, Q61, Q79, Q80, Q81, Q897, Q9
Obesity if over the age of 16 years	E66
Pregnancy (only considered up to nine months prior to appearance in cohort)	O
**Medical and developmental UHC (a wider selection based on consultant paediatrician views)**	**ICD-10 Codes**
Cancer and Neoplasms excluding benign tumours, (only considered within the prior 5 years)	C, D0, D37, D38, D39, D4
Blood Disorders and Immune Deficiencies	B20, B21, B22, B24, D55, D56, D57, D58, D59, D60, D61, D64.0, D64.4, D66, D67, D68.0, D68.1, D68.2, D70, D71, D72.0, D73.0, D73.1, D76.1, D76.3, D8, R16
Endocrine Conditions	E00, E10, E11, E12, E13, E14, E20, E21, E22, E23, E24, E25, E26, E27, E3, E4, E6, E7, E80, E83, E84, E85, E88, E89
Neurological and Developmental Conditions	F72, F73, F78, F79, F83, F84, F88, F89, G1, G20, G21, G22, G23, G24, G3, G40, G45, G47, G5, G6, G7, G8, G91, G94, P21, Q0, Q1, Q90, Q91, Q92, Q93, R56.8, R62, R94
Respiratory Conditions	B27 (only considered in the prior 5 years), J38.0, J38.6, J41, J42, J43, J44, J45, J46, J47, J82, J84, J98.4, J99, Q30, Q31, Q32, Q33, Q34

Congenital Heart Disease, Hypertension and Acquired Heart disease	I05, I06, I07, I08, I09, I10, I11, I12, I13, I15, I27, I28, I31, I34, I35, I36, I37, I42, I43, I44, I45, I47, I48, I49, I50, I51, I52, Q2
Digestive and Liver Conditions	K44, K50, K51, K71, K72.1, K74.0, K74.1, K74.4, K74.5, K74.6, K75, K76, K90, Q35, Q36, Q37, Q39, Q4
Muscle, Skin and Arthritis	L1, L85, L93, L94, M05, M06, M07, M08, M09, M30.0, M30.1, M30.2, M31, M32.1, M32.8, M32.9, M34, M35, M41, M42, M43, Q65, Q67, Q71, Q72, Q73, Q74, Q75, Q76, Q77, Q78, Q79, Q80, Q81, Q85, Q87, Q89
Renal and Genitourinary Conditions	N0, N11, N12, N13, N15, N18, N31, N32, Q5, Q60, Q61, Q62, Q63, Q64
Prematurity and Low Birth Weight, (only considered within the prior 5 years)	P05, P07

Table C: Characteristics of the ICU or HDU admitted subset of all first SARS-CoV-2 associated hospitalisations, captured amongst first infections in our cohort, as well as stratification by hospitalisation type

		Admission Type						
					Caused by SARS-CoV-2		SARS-CoV-2 on Causal Pathway	
	Overall	Nosocomial	Type C (Incidental)	PIMS-TS	Type A1 (Caused)	Type A2 (Suspected)	Type B1 (Contributed)	Type B2 (Contributed)
**Number**	1,710	105	255	535	315	95	320	85
**Sex**								
Female	775 (45.3)	45 (42.9)	120 (47.1)	215 (40.2)	160 (50.8)	45 (47.4)	150 (46.9)	40 (47.1)
Male	940 (55.0)	60 (57.1)	135 (52.9)	320 (59.8)	160 (50.8)	50 (52.6)	175 (54.7)	45 (52.9)
**AGE (median [IQR])**	5.42 [0.22, 11.19]	0.69 [0.23, 2.06]	2.58 [0.07, 10.58]	9.74 [6.78, 12.23]	4.18 [0.38, 12.48]	1.92 [0.16, 9.50]	0.09 [0.04, 4.68]	2.22 [0.06, 10.15]
**AGE Group**								
< 1	565 (33.1)	60 (57.1)	115 (45.1)	14 (2.7)	95 (30.2)	35 (36.8)	205 (64.1)	35 (41.2)
1 - 4	265 (15.5)	30 (28.6)	40 (15.7)	60 (11.2)	65 (20.6)	20 (21.1)	40 (12.5)	10 (11.8)
5 - 11	535 (31.3)	<10 (<9.5)	45 (17.6)	320 (59.9)	70 (22.2)	25 (26.3)	45 (14.1)	20 (23.5)
12 - 15	305 (17.8)	<10 (<9.5)	50 (19.6)	125 (23.4)	70 (22.2)	15 (15.8)	25 (7.8)	15 (17.6)
16 - 17	40 (2.3)	0 (0.0)	<10 (<3.9)	15 (2.8)	15 (4.8)	<10 (<10.5)	<10 (<3.1)	<10 (<11.8)
**Ethnic Group**								
Asian or Asian British	280 (16.4)	15 (14.3)	35 (13.7)	115 (21.5)	50 (15.9)	15 (15.8)	40 (12.5)	<10 (<11.8)
Black or Black British	195 (11.4)	10 (9.5)	15 (5.9)	95 (17.8)	40 (12.7)	<10 (<10.5)	25 (7.8)	<10 (<11.8)
Chinese	10 (0.6)	0 (0.0)	<10 (<3.9)	<10 (<1.9)	<10 (<3.2)	0 (0.0)	<10 (<3.1)	0 (0.0)
Mixed	100 (5.8)	<10 (<9.5)	15 (5.9)	35 (6.5)	20 (6.3)	<10 (<10.5)	15 (4.7)	<10 (<11.8)
Other	55 (3.2)	<10 (<9.5)	<10 (<3.9)	15 (2.8)	10 (3.2)	<10 (<10.5)	20 (6.2)	<10 (<11.8)
Unknown	15 (0.9)	<10 (<9.5)	<10 (<3.9)	<10 (<1.9)	<10 (<3.2)	0 (0.0)	<10 (<3.1)	<10 (<11.8)
White	1,055 (61.7)	70 (66.7)	180 (70.6)	270 (50.5)	195 (61.9)	65 (68.4)	220 (68.8)	65 (76.5)
**IMD Quintile**								
1 (most deprived)	500 (29.2)	35 (33.3)	80 (31.4)	140 (26.2)	105 (33.3)	25 (26.3)	95 (29.7)	25 (29.5)
2	405 (23.7)	20 (19.0)	55 (21.6)	120 (22.4)	80 (25.4)	30 (31.6)	75 (23.4)	15 (17.6)
3	315 (18.4)	25 (23.8)	40 (15.7)	100 (18.7)	50 (15.9)	20 (21.1)	60 (18.8)	20 (23.5)
4	265 (15.5)	20 (19.0)	45 (17.6)	90 (16.8)	40 (12.7)	10 (10.5)	55 (17.2)	10 (11.8)

5 (least deprived)	225 (13.2)	<10 (<9.5)	35 (13.7)	85 (15.9)	40 (12.7)	10 (10.5)	35 (10.9)	15 (17.6)
**Presence of a health condition linked to clinical vulnerability**	835 (48.8)	80 (76.2)	100 (39.2)	185 (34.6)	185 (58.7)	50 (52.6)	165 (51.6)	60 (70.6)
**Presence of M-D Risk Factor**	1,320 (77.2)	100 (95.2)	165 (64.7)	355 (66.4)	260 (82.5)	70 (73.7)	285 (89.1)	85 (100.0)
**COVID Positive in Window***	1,070 (62.6)	105 (100.0)	225 (88.2)	60 (11.2)	265 (84.1)	80 (84.2)	260 (81.2)	75 (88.2)
**Length of Stay**	7.00 [4.00, 13.00]	87.00 [47.75, 148.25]	8.00 [4.00, 15.00]	6.00 [4.00, 9.00]	6.00 [3.00, 11.00]	4.00 [2.00, 9.25]	9.00 [4.00, 29.00]	8.50 [3.25, 13.00]
**Still in Hospital**	45 (2.6)	15 (14.3)	15 (5.9)	0 (0.0)	<10 (<3.2)	<10 (<10.5)	<10 (<3.1)	<10 (<11.8)
**Day Case**	<10 (<0.6)	0 (0.0)	0 (0.0)	<10 (<1.9)	0 (0.0)	0 (0.0)	0 (0.0)	<10 (<11.8)
**Vaccination Status 14 Days Prior to Infection**								
Unvaccinated	1,690 (98.8)	105 (100.0)	255 (100.0)	530 (99.1)	315 (100.0)	95 (100.0)	315 (98.4)	85 (100.0)
First Dose	15 (0.9)	<10 (<9.5)	<10 (<3.9)	<10 (<1.9)	<10 (<3.2)	0 (0.0)	<10 (<3.1)	0 (0.0)
Second Dose	<10 (<0.6)	0 (0.0)	0 (0.0)	0 (0.0)	<10 (<3.2)	0 (0.0)	<10 (<3.1)	<10 (<11.8)
Booster Dose	0 (0.0)	0 (0.0)	0 (0.0)	0 (0.0)	0 (0.0)	0 (0.0)	0 (0.0)	0 (0.0)
**Variant Period**								
Original	165 (9.6)	15 (14.3)	25 (9.8)	45 (8.4)	30 (9.5)	<10 (<10.5)	40 (12.5)	<10 (<11.8)
Inter-Original-Alpha	125 (7.3)	<10 (<9.5)	15 (5.9)	35 (6.5)	20 (6.3)	<10 (<10.5)	35 (10.9)	<10 (<11.8)
Alpha	325 (19.0)	20 (19.0)	30 (11.8)	165 (30.8)	55 (17.5)	15 (15.8)	35 (10.9)	<10 (<11.8)
Inter-Alpha-Delta	20 (1.2)	0 (0.0)	<10 (<3.9)	<10 (<1.9)	<10 (<3.2)	<10 (<10.5)	<10 (<3.1)	<10 (<11.8)
Delta	675 (39.5)	30 (28.6)	95 (37.3)	215 (40.2)	140 (44.4)	30 (31.6)	130 (40.6)	35 (41.2)
Inter-Delta-Omicron	65 (3.8)	<10 (<9.5)	10 (3.9)	20 (3.7)	10 (3.2)	0 (0.0)	20 (6.2)	<10 (<11.8)
Omicron	335 (19.6)	30 (28.6)	75 (29.4)	55 (10.3)	60 (19.0)	30 (31.6)	60 (18.8)	30 (35.3)
**Death**	60 (3.5)	<10 (<9.5)	10 (3.9)	0 (0.0)	20 (6.3)	<10 (<10.5)	15 (4.7)	<10 (<11.8)
**Hospital Death**	35 (2.0)	<10 (<9.5)	<10 (<3.9)	0 (0.0)	10 (3.2)	<10 (<10.5)	10 (3.1)	<10 (<11.8)
**COVID or PIMS-TS Related Death**	25 (1.5)	<10 (<9.5)	<10 (<3.9)	0 (0.0)	15 (4.8)	<10 (<10.5)	<10 (<3.1)	<10 (<11.8)
**COVID or PIMS-TS Underlying Death**	15 (0.9)	0 (0.0)	<10 (<3.9)	0 (0.0)	10 (3.2)	0 (0.0)	<10 (<3.1)	0 (0.0)
**COVID or PIMS-TS Related Hospital Death**	20 (1.2)	<10 (<9.5)	<10 (<3.9)	0 (0.0)	10 (3.2)	<10 (<10.5)	<10 (<3.1)	<10 (<11.8)
**COVID or PIMS-TS Underlying Hospital Death**	10 (0.6)	0 (0.0)	0 (0.0)	0 (0.0)	<10 (<3.2)	0 (0.0)	<10 (<3.1)	0 (0.0)

*Counted only when the test occurs in the period of 14 days before date of admission up to discharge, this window defines an admission as SARS-CoV-2-related, and it could be (but equally may not be the sole) reason for their inclusion in this part of the cohort.

Table D: Risk factors linked to clinical vulnerability, underlying health conditions selected by paediatricians, pregnancy, and obesity evidence rates by vaccination status for children who had first ascertained infection after 19th July 2021 and their 12th birthday

We show the counts of "vaccinated" (signifies any doses received) versus "unvaccinated" children where their first ascertained infection occurred after July 19th 2021 (when the use of Pfizer-BioNTech Covid-19 vaccine was approved for use in vulnerable 12- to 15-year-olds) restricted to children over 12 years old only.

	First Ascertained SARS-CoV-2 Infections						
		Via Positive SARS-CoV-2 Test Only		Via COVID-Related Hospital Admission Within 6 Weeks of Infection		ICU / HDU Subset	
	Total	Unvaccinated	Vaccinated	Unvaccinated	Vaccinated	Unvaccinated	Vaccinated
**Number**	1,133,155	782,490	345,250	4,050	1,365	160	20
**Presence of a Health Condition Linked to Greater Vulnerability**	232,425 (20.5)	151,860 (19.4)	77,735 (22.5)	2,010 (49.6)	825 (60.4)	95 (59.4)	15 (75.0)
Cancer (Excluding Benign Tumours)	1,460 (0.1)	745 (0.1)	525 (0.2)	120 (3.0)	70 (5.1)	<10 (<6.2)	<10 (<50.0)
Blood Disorders and Immune Deficiencies	4,480 (0.4)	2,675 (0.3)	1,500 (0.4)	210 (5.2)	95 (7.0)	<10 (<6.2)	<10 (<50.0)
Endocrine Conditions	8,990 (0.8)	5,300 (0.7)	3,310 (1.0)	270 (6.7)	110 (8.1)	20 (12.5)	<10 (<50.0)
Severe Neurological and Developmental Conditions	49,380 (4.4)	31,540 (4.0)	17,085 (4.9)	520 (12.8)	240 (17.6)	35 (21.9)	<10 (<50.0)
Hypertension, Cardiac Valves and Cardiomyopathy	3,925 (0.3)	2,230 (0.3)	1,440 (0.4)	160 (4.0)	95 (7.0)	30 (18.8)	<10 (<50.0)
Severe Respiratory Diseases	162,880 (14.4)	107,890 (13.8)	53,735 (15.6)	890 (22.0)	365 (26.7)	25 (15.6)	<10 (<50.0)
Digestive, Liver and Renal Diseases	3,165 (0.3)	1,580 (0.2)	1,260 (0.4)	185 (4.6)	135 (9.9)	<10 (<6.2)	<10 (<50.0)
Arthritis and Connective Tissue Diseases	3,895 (0.3)	2,375 (0.3)	1,385 (0.4)	80 (2.0)	60 (4.4)	<10 (<6.2)	0 (0.0)
Congenital Syndromes and Anomalies	20,725 (1.8)	12,890 (1.6)	7,345 (2.1)	305 (7.5)	185 (13.6)	30 (18.8)	<10 (<50.0)
Obesity (>16 Years of Age)	4,310 (0.4)	2,055 (0.3)	2,140 (0.6)	85 (2.1)	35 (2.6)	<10 (<6.2)	0 (0.0)
Recent or current Pregnancy	2,370 (0.2)	1,415 (0.2)	730 (0.2)	195 (4.8)	30 (2.2)	0 (0.0)	0 (0.0)

**M-D Underlying Health Conditions**	329,770 (29.1)	219,645 (28.1)	106,775 (30.9)	2,405 (59.4)	945 (69.2)	120 (75.0)	20 (100.0)
Cancer and Neoplasms (Excluding Benign Tumours)	1,840 (0.2)	990 (0.1)	645 (0.2)	130 (3.2)	75 (5.5)	<10 (<6.2)	<10 (<50.0)
Blood Disorders and Immune Deficiencies	8,600 (0.8)	5,165 (0.7)	2,900 (0.8)	365 (9.0)	170 (12.5)	15 (9.4)	<10 (<50.0)
Endocrine Conditions	45,285 (4.0)	29,470 (3.8)	14,780 (4.3)	740 (18.3)	295 (21.6)	45 (28.1)	<10 (<50.0)
Neurological and Developmental Conditions	96,770 (8.5)	63,605 (8.1)	31,700 (9.2)	1,030 (25.4)	435 (31.9)	60 (37.5)	10 (50.0)
Respiratory Conditions	176,980 (15.6)	117,395 (15.0)	58,115 (16.8)	1,040 (25.7)	430 (31.5)	40 (25.0)	10 (50.0)
Congenital Hypertension and Heart Disease (Congenital and Acquired)	143,305 (12.6)	93,575 (12.0)	48,420 (14.0)	910 (22.5)	400 (29.3)	70 (43.8)	15 (75.0)
Digestive and Liver Conditions	15,445 (1.4)	9,435 (1.2)	5,550 (1.6)	275 (6.8)	185 (13.6)	10 (6.2)	<10 (<50.0)
Muscle, Skin and Arthritis	20,515 (1.8)	12,840 (1.6)	7,160 (2.1)	340 (8.4)	175 (12.8)	20 (12.5)	<10 (<50.0)
Renal and Genitourinary Conditions	32,780 (2.9)	21,975 (2.8)	10,370 (3.0)	300 (7.4)	135 (9.9)	20 (12.5)	<10 (<50.0)
Prematurity and Low Birth Weight	45 (0.0)	25 (0.0)	20 (0.0)	<10 (<0.2)	0 (0.0)	0 (0.0)	0 (0.0)
**Evidence of Obesity in EHR**	35,555 (3.1)	23,870 (3.1)	11,150 (3.2)	410 (10.1)	125 (9.2)	25 (15.6)	<10 (<50.0)
**Recent or current Pregnancy**	2,370 (0.2)	1,415 (0.2)	730 (0.2)	195 (4.8)	30 (2.2)	0 (0.0)	0 (0.0)

Table E: Number of first admissions by type and ascertained participant hospitalisation rates for each variant wave amongst only those children and adolescents that had a positive test

We display total infections, admissions (by type) and the resulting hospital admission rates in parentheses by variant wave. Inter-wave period data is not shown. Each wave's hospital admission rate is calculated as the percentage of first infections starting in each period that led to (a) all SARS-CoV-2-associated admissions; (b) Type A admissions; (c) Type B admissions; and (d) admissions involving an ICU or HDU stay.

	**Variant Period**			
	**Original** 1st Jul 2020 to 5th Dec 2020	**Alpha** 3rd Jan 2021 to 1st May 2021	**Delta** 30th May 2021 to 11th Dec 2021	**Omicron** 26th Dec 2021 to 17th Feb 2022
**Number of First Ascertained Infections**	126,745	158,280	1,611,125	1,013,300
**COVID-Related Hospital Admission Type**				
All types (%)	1,950 (1.5)	2,565 (1.6)	9,180 (0.6)	7,520 (0.7)
Type A (caused or suspectedly caused by SARS-CoV-2) (%)	940 (0.7)	1,320 (0.8)	5,075 (0.3)	4,100 (0.4)
Type B (SARS-CoV-2 on causal pathway) (%)	300 (0.2)	335 (0.2)	1,320 (0.1)	1,130 (0.1)
ICU or HDU care (%)	120 (0.1)	150 (0.1)	415 (0.0)	255 (0.0)

Table F: The percentage prevalence of each definitive code for each type of hospitalisation

Patients may contribute to more than one column so for example, if a patient had a U07.2 and U07.4 code, they contribute to both the U07.2 and U07.4 columns.

Type of Admission	Hospitalisations with Code U07.1 (%)	Hospitalisations with Code U07.2 (%)	Hospitalisations with Code U07.3 (%)	Hospitalisations with Code U07.4 (%)	Hospitalisations with no Covid Codes and thus only a Positive Test or PIMS-TS code (%)
**Nosocomial**	19.8	0.0	2.6	0.0	78.6
**Incidental**	63.5	1.3	4.7	0.2	30.7
**PIMS-TS**	8.5	4.1	7.7	0.6	79.3
**Type A1**	96.9	2.8	0.2	0.2	0.0
**Type A2**	54.3	3.7	7.4	0.8	33.9
**Type B1**	69.4	4.7	3.2	0.2	22.8
**Type B2**	52.0	2.4	5.7	0.3	39.7

Type of Admission	Hospitalisations with Code U07.5 (%)	Hospitalisations with Code M30.3 (%)	Hospitalisations with Code R65 (%)
**PIMS-TS**	60.6	37.0	8.3

Supplementary Materials Wilde et al 7th June 2023

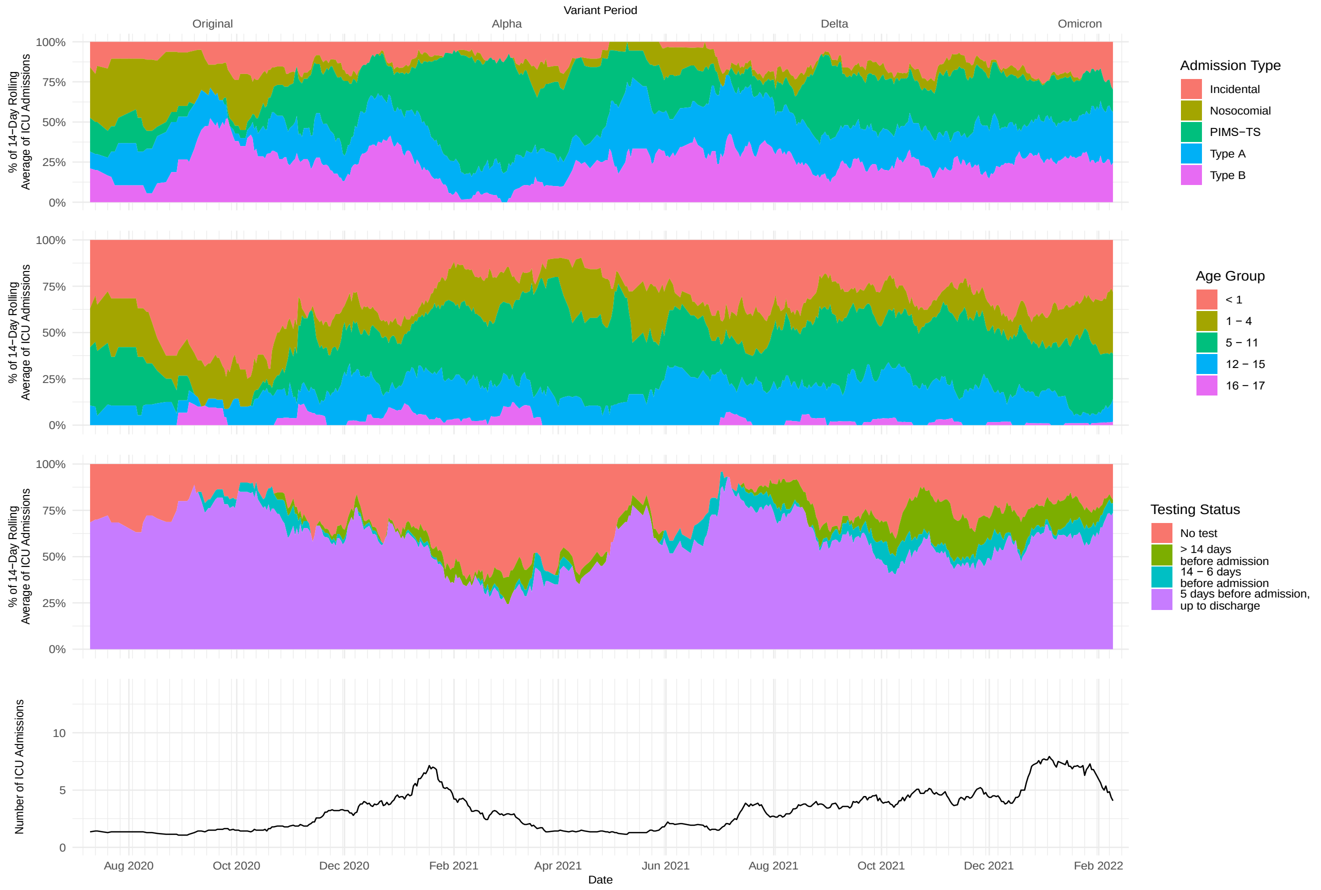

Figure A: SARS-CoV-2 Associated ICU or HDU Hospitalisation Characteristics Over Time

The first three panels illustrate proportional changes in the rolling average of first SARS-CoV-2 associated ICU/HDU admissions across three different stratifications of the cohort, (1) by hospitalisation type, (2) by age group and (3) by SARS-CoV-2 testing status of participants, i.e. whether there was evidence of a Positive SARS-CoV-2 test alongside hospitalisation and when the test was dated. The bottom part of this figure shows the daily count of hospitalisations throughout the study period, with the black line indicating the 14-day rolling average of these counts.

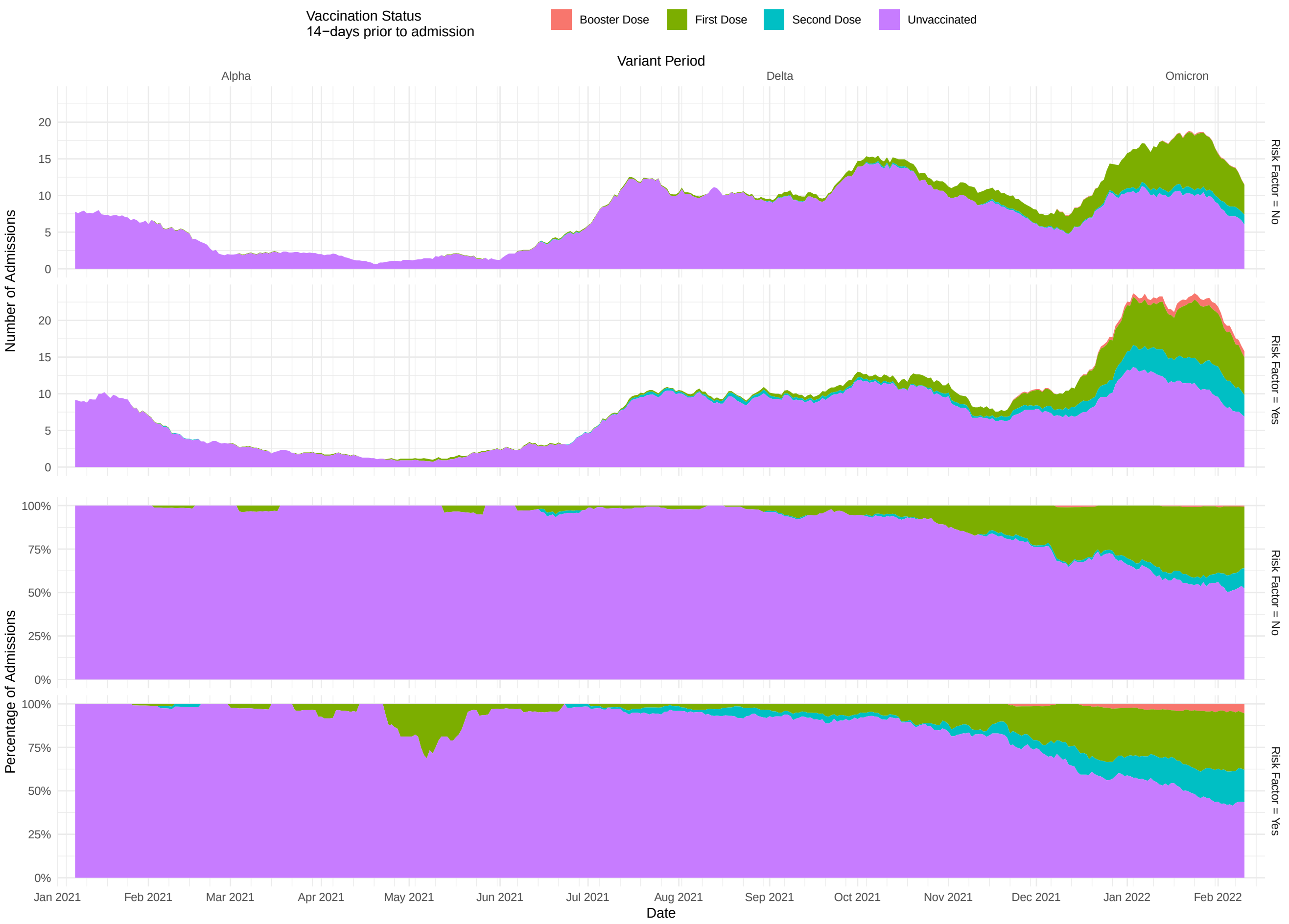

Figure B: Vaccination Status Amongst Children Over the Age of 12 years who had a SARS-CoV-2 Associated Hospitalisation

This figure shows the number (top two panels) and percentage (bottom two panels) of the 14-day rolling average of hospital admissions occurring since the start of 2021, with an additional stratification by evidence of a risk factor associated with greater clinical vulnerability in Chapter 14a of the Green Book, and illustrating the breakdown of vaccinated, first dose, second dose and booster dose populations.

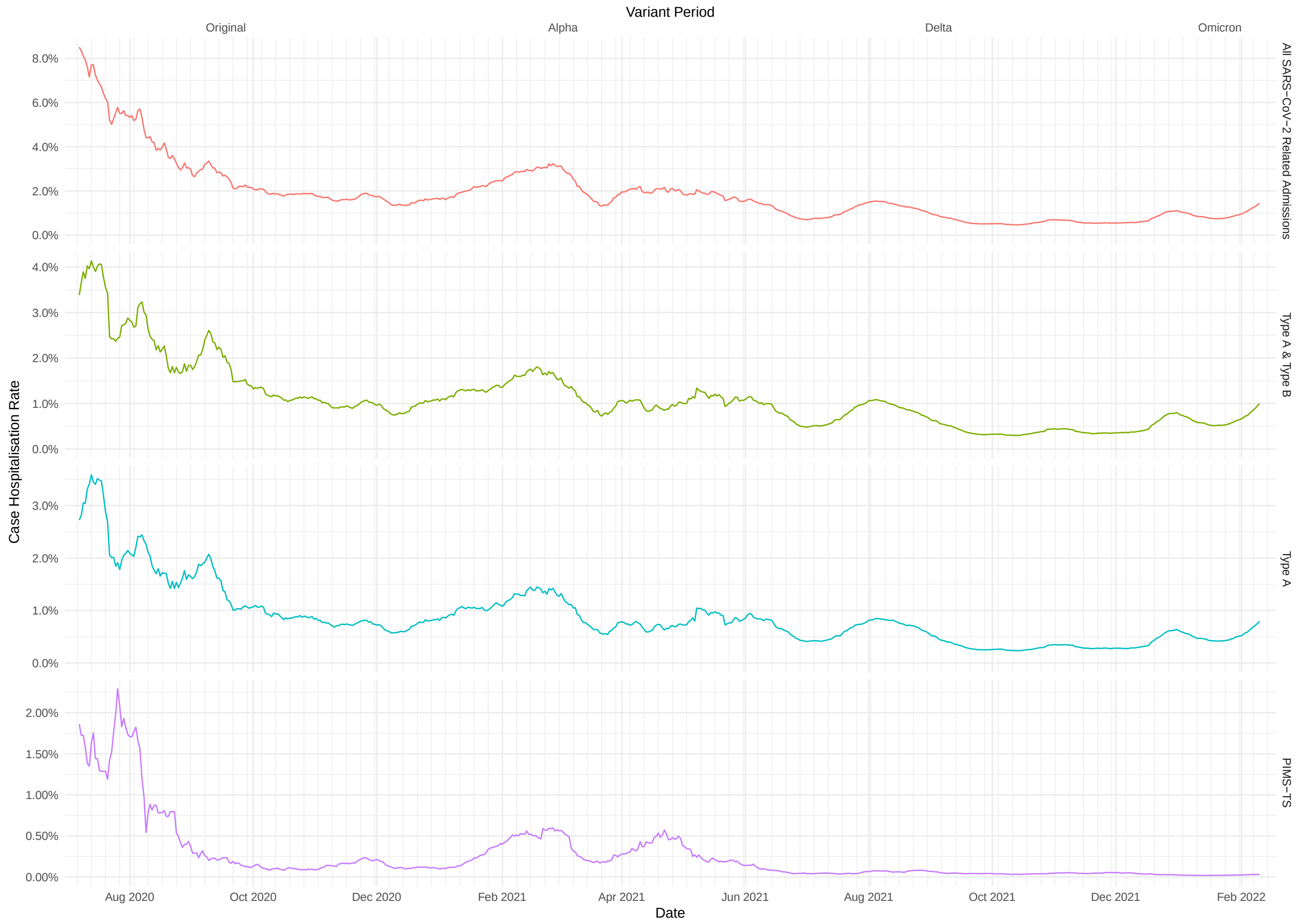

Figure C: Ascertained Hospitalisation Rates for Different Hospitalisation Types

We show the 14-day rolling average of the daily hospital admission rate for all hospitalisations in our cohort, as well as Type A and B, Type A only, and PIMS-TS subsets of the cohort. We note that case ascertainment and testing capacity / policy strongly affect these values, as we discuss in the manuscript. Note that the y-axis limits vary for each panel in this figure to best illustrate the time dynamics of the hospital admission rate.
